# Positive roles of the Ca^2+^ sensors GbCML45 and GbCML50 in improving cotton Verticillium wilt resistance

**DOI:** 10.1111/mpp.13483

**Published:** 2024-06-03

**Authors:** Feifei Yi, Yuzhe Li, Aosong Song, Xinying Shi, Shanci Hu, Shuang Wu, Lili Shao, Zongyan Chu, Kun Xu, Liangliang Li, Lam‐Son Phan Tran, Weiqiang Li, Yingfan Cai

**Affiliations:** ^1^ National Key Laboratory of Cotton Biological Breeding and Utilization, School of Life Sciences Sanya Institute, Henan University Kaifeng China; ^2^ Jilin Da'an Agro‐Ecosystem National Observation Research Station, Changchun Jingyuetan Remote Sensing Experiment Station, State Key Laboratory of Black Soils Conservation and Utilization, Northeast Institute of Geography and Agroecology Chinese Academy of Sciences Changchun China; ^3^ Department of Plant and Soil Science, Institute of Genomics for Crop Abiotic Stress Resistance Texas Tech University Lubbock Texas USA

**Keywords:** calmodulin, cotton, hormones, reactive oxygen species, Verticillium wilt resistance

## Abstract

As a universal second messenger, cytosolic calcium (Ca^2+^) functions in multifaceted intracellular processes, including growth, development and responses to biotic/abiotic stresses in plant. The plant‐specific Ca^2+^ sensors, calmodulin and calmodulin‐like (CML) proteins, function as members of the second‐messenger system to transfer Ca^2+^ signal into downstream responses. However, the functions of CMLs in the responses of cotton (*Gossypium* spp.) after *Verticillium dahliae* infection, which causes the serious vascular disease Verticillium wilt, remain elusive. Here, we discovered that the expression level of *GbCML45* was promoted after *V. dahliae* infection in roots of cotton, suggesting its potential role in Verticillium wilt resistance. We found that knockdown of *GbCML45* in cotton plants decreased resistance while overexpression of *GbCML45* in *Arabidopsis thaliana* plants enhanced resistance to *V. dahliae* infection. Furthermore, there was physiological interaction between GbCML45 and its close homologue GbCML50 by using yeast two‐hybrid and bimolecular fluorescence assays, and both proteins enhanced cotton resistance to *V. dahliae* infection in a Ca^2+^‐dependent way in a knockdown study. Detailed investigations indicated that several defence‐related pathways, including salicylic acid, ethylene, reactive oxygen species and nitric oxide signalling pathways, as well as accumulations of lignin and callose, are responsible for *GbCML45*‐ and *GbCML50*‐modulated *V. dahliae* resistance in cotton. These results collectively indicated that GbCML45 and GbCML50 act as positive regulators to improve cotton Verticillium wilt resistance, providing potential targets for exploitation of improved Verticillium wilt‐tolerant cotton cultivars by genetic engineering and molecular breeding.

## Graphical abstract

The Ca^2+^ sensors GbCML45 and GbCML50 enhance cotton Verticillium wilt resistance by stimulating reactive oxygen species burst, NO accumulation, deposition of lignin and callose, and salicylic acid and ethylene signalling pathways.

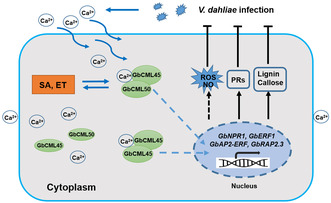

## 1 INTRODUCTION

Calcium (Ca^2+^) works as a second messenger participating in signal transduction to regulate plant growth, development and multi‐environmental responses through regulation of various cellular processes (Berridge et al., 2000; Sanders et al., 1999). The variation of cellular Ca^2+^ levels in plants shows spatiotemporal properties according to various environmental stimuli and stress conditions (Aldon et al., 2018; Bender & Snedden, 2013). In plants, the rapid movement of Ca^2+^ into the cytosol is a key event activated by the perception of pathogen‐associated molecular patterns (PAMPs) such as during the early stage of pathogen attack, or by perception of abiotic stresses such as drought or exposure to ozone (Köster et al., 2022; Kudla et al., 2018; Lecourieux et al., 2006). To perceive a stimulus, the Ca^2+^ messenger is decoded and relayed by Ca^2+^‐binding proteins (also named Ca^2+^ sensors) to activate cellular responses (Steinhorst & Kudla, 2014). Four groups of Ca^2+^ sensors have been identified in plants: calmodulin (CaM), calmodulin‐like (CML), calcineurin B‐like (CBL) and the Ca^2+^‐dependent protein kinase (CDPK/CPK) proteins (Batistič & Kudla, 2012; McCormack et al., 2005; Zhu et al., 2015). Many Ca^2+^ sensors possess a highly conserved helix–loop–helix motif (also named EF‐hand) that contains 29 amino acids and forms a loop structure; the 12 central residues can bind one Ca^2+^ ion (La Verde et al., 2018). The expression patterns of *CML*s are regulated by environmental stimuli or various plant developmental stages, indicating that CMLs may play specific roles in growth, development and different stress responses in plant (Cheval et al., 2013; Ranty et al., 2016; Zhu et al., 2015).

CaMs/CMLs are involved in plant immunity by activating CaM/CML‐binding transcription factors (TFs) and regulating plant defence in several plant species (Du et al., 2009; Galon et al., 2008; Wang et al., 2009, 2011). CaMs/CMLs also target a large group of downstream CaM‐binding proteins (CBPs), including transporters, protein phosphatases, protein kinases and metabolic enzymes, and form complexes in Ca^2+^‐dependent or ‐independent ways (Reddy et al., 2011; Zeng et al., 2015, 2023). Several members of CMLs in *Arabidopsis thaliana*, such as CML8, CML9, CML41 and CML43, have been shown to positively regulate plant resistance to *Pseudomonas syringae* pv. *tomato* (Pst) DC3000 (Chiasson et al., 2005; Leba et al., 2012; Xu et al., 2017; Zhu et al., 2017). *CML46* and *CML47* also modulate salicylic acid (SA) accumulation and enhance *Arabidopsis* resistance to the pathogen *P. syringae* pv. *maculicola* (Pma) (Lu et al., 2018). CML orthologues positively regulating plant disease resistance have also been discovered in other plant species. For example, overexpression of soybean (*Glycine max*) *SCaM4* and *SCaM5* genes in *Arabidopsis* promotes the resistance of transgenic plants to *P. syringae* and *Phytophthora sojae* (Heo et al., 1999; Park et al., 2004). Silencing the pathogen‐induced *NtCaM13*, an orthologue of *Arabidopsis CaM4* and *CaM5*, in *Nicotiana benthamiana* results in elevated susceptibility of plants to necrotrophic pathogens such as *Alternaria tenuissima* and *Phomopsis longicolla* (Takabatake et al., 2007). In addition, wheat (*Triticum aestivum*) *TaCML36* and pepper (*Capsicum annuum*) *CaCML13* also enhance immune responses to *Rhizoctonia cerealis* (Lu et al., 2019) and *Ralstonia solanacearum* in plants (Shen et al., 2020), respectively. However, tomato (*Solanum lycopersicum*) SlCML55 decreased defence against *Phytophthora* pathogens by inhibiting the SA signalling pathway (Zhang, Zou, et al., 2022). These studies suggest that different *CaM* and *CML* genes may function as positive or negative regulators of pathogen resistance in different plant species.

Cotton (*Gossypium* spp.) Verticillium wilt is a serious vascular disease that is caused by the fungus *Verticillium dahliae*, resulting in significant economic losses for cotton producers worldwide (Cai et al., 2009; Sal'kova & Guseva, 1965). To resist *Verticillium* infection, cotton has evolved multiple defence mechanisms, including reinforcement of cell wall structure, maintenance of reactive oxygen species (ROS) homeostasis and activation of mitogen‐activated protein kinase (MAPK) cascades, hormone signalling and PAMP‐/effector‐triggered immunity (PTI/ETI) responses (Shaban et al., 2018; Umer et al., 2023; Zhu et al., 2023). Overexpression of *respiratory burst oxidase homologue D* (*GhRbohD*), *GbRboh5* or *GbRboh18*, in cotton promotes the resistance of transgenic plants to *V. dahliae* by inducing accumulation of ROS or nitric oxide (NO) (Chang et al., 2020; Huang et al., 2021). Enhancing cell wall lignification through increasing the biosynthesis of lignin also promotes cotton resistance to *V. dahliae* (Guo et al., 2016; Shi et al., 2012; Xu et al., 2011; Zhang et al., 2019; Zhu et al., 2018). For example, overexpression of genes encoding rate‐limiting enzymes of lignin biosynthesis or *ethylene responsive transcription factor 1‐like (GbERF1‐like)* gene in cotton plants led to an increased lignin accumulation and enhanced *V. dahliae* resistance of cotton plants (Guo et al., 2016; Sun et al., 2013; Xu et al., 2011). This finding also indicates that ethylene (ET) signalling functions in plant resistance to *V. dahliae* infection (Jia et al., 2022; Wang et al., 2023). Other phytohormones, including jasmonic acid (JA), SA and strigolactones (SLs), also participate in plant resistance to *Verticillium* infection (Jia et al., 2022; Song et al., 2020; Yi et al., 2023). JA enhances plant resistance to *V. dahliae* infection by stimulating the downstream *myelocytomatosis 2* (*MYC2*) and *plant defence factor 1.2* (*PDF1.2*) gene expression (He et al., 2018). The SA‐regulated defence pathway displays both cooperative and inhibitory effects on JA signalling pathway (Wang et al., 2020). The non‐expressor of pathogenesis‐related protein 1 (NPR1) and NPR1‐like protein 3 (NPR3)/NPR4 receptors perceive the SA‐mediated responses to prompt the expression of *pathogenesis‐related* (*PR*) genes, such as *PR1* and *PR5*, which in turn increases plant resistance against *V. dahliae* infection in some plant species, including cotton (Liu et al., 2020; Wu et al., 2012; Yan et al., 2016). On the other hand, the suppression of GhWRKY70A05a, a negative regulator of cotton resistance to *V. dahliae*, improved cotton resistance to *V. dahliae* infection by inhibiting the SA signalling pathway and promoting the JA signalling pathway (Xiong et al., 2019). Genetic and breeding studies have indicated that numerous TF‐ and enzyme‐encoding genes, such as *polygalacturonase‐inhibiting protein* (*GhPGIP1*), *major latex protein* (*GhMLP28*), *GbWRKY1*, *GbCAD1*, *laccase* (*GhLAC15*), *GhMKK4/6/9* and *pectin methylesterases* (*GhPMEI13*), are involved in *V. dahliae* resistance in cotton (Gao et al., 2013; Li et al., 2014, 2018; Liu et al., 2017; Meng et al., 2018; Yang et al., 2015; Zhang et al., 2019; Zhang, Liu, et al., 2022).

Recently, some Ca^2+^‐dependent proteins were reported to be associated with cotton Verticillium wilt resistance. For example, the knockdown of *GhCPK33* led to decreased resistance to *V. dahliae* infection by the down‐regulation of JA biosynthesis in cotton (Hu et al., 2018), while the myeloblastosis 108 (MYB108) TF forms a positive feedback loop to promote the transcription of *GhCML11* in a Ca^2+^‐dependent way in enhancing *V. dahliae* resistance (Cheng et al., 2016). Mutation in plant‐specific master TF‐encoding *CBP60g* or *systemic acquired resistance‐deficient 1* (*SARD1*), which modulate many defence‐related genes in immunity and are targeted by a *V. dahliae* secretory protein 41 (VdSCP41), caused compromised resistance of *Arabidopsis* mutants against *V. dahliae* (Qin et al., 2018). Moreover, silencing of *GhCBP60b*, also a target of VdSCP41, compromised resistance to *V. dahliae* in cotton (Qin et al., 2018). Acetylation of GhCaM7 enhances resistance to *V. dahliae* through increasing its interaction with the osmotin protein GhOSM34 in cotton, which then induces multiple disease resistance signalling pathways (Zhang et al., 2023). However, the mechanisms Ca^2+^/CaM/CML‐regulated pathways underlying Verticillium wilt resistance remain unknown in cotton.

In this study, by analysing root transcriptome data, we identified a *V. dahliae*‐responsive gene, *GbCML45*, from roots of the island cotton (*Gossypium barbadense*) and found that it positively regulated Verticillium wilt resistance in both cotton and *Arabidopsis*. We found that GbCML45 can interact with its close homologue GbCML50. Further investigations revealed that *GbCML50* also has positive regulatory role in Verticillium wilt resistance in cotton, where both *GbCML45* and *GbCML50* genes modulate the defence‐related SA and ET signalling pathways, ROS burst, lignin synthesis and callose deposition to enhance cotton resistance to *V. dahliae* infection.

## 2 RESULTS

### 2.1 *V. dahliae* infection induces the expression of *GbCML45* in cotton and knockdown of *GbCML45* decreases cotton resistance to Verticillium wilt

**FIGURE 1:**
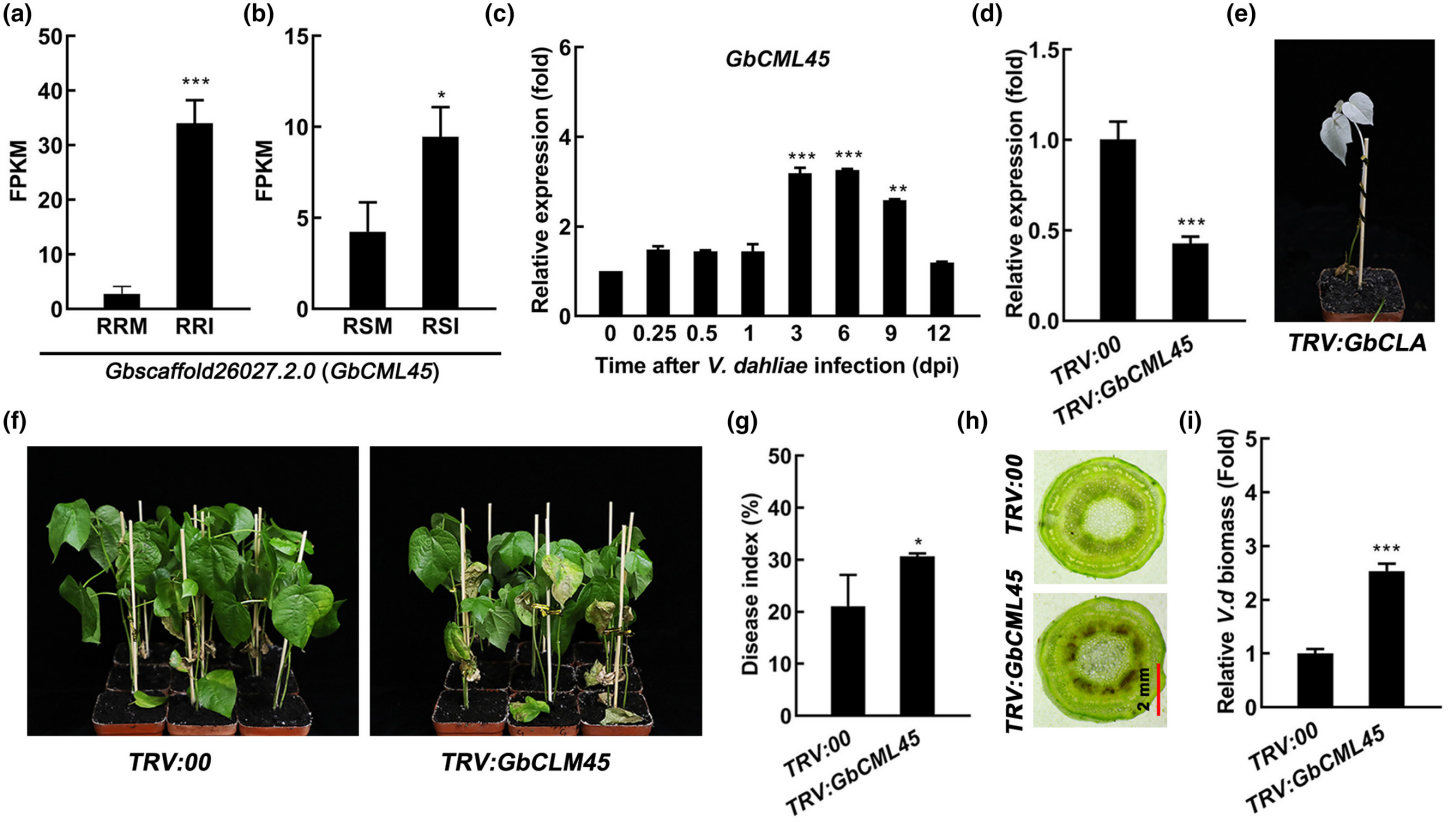
*Verticillium dahliae* infection induces the expression of *GbCML45* in roots of cotton, and silencing of *GbCML45* reduces resistance to *V. dahliae* in cotton. (a) and (b) The fragments per kilobase of transcript per million mapped reads (FPKM) values of *Gbscaffold26027.2.0* (*GbCML45*) in roots of resistant cultivar CSSL1 and susceptible cultivar CSSL4 of cotton. (a) Root‐resistant cultivar mock (RRM) and root‐resistant cultivar inoculated (RRI). (b) Root‐susceptible cultivar mock (RSM) and root‐susceptible cultivar inoculated (RSI). (c) The transcript levels of *GbCML45* in the roots of island cotton (Hai7124) after 0, 0.25, 0.5, 1, 3, 5, 9 and 12 days post‐inoculation (dpi) with *V. dahliae* infection. (d) The relative transcript levels of *GbCML45* in roots of *TRV:GbCML45* cotton plants. (e) Albino phenotype of the cotton plants inoculated with *TRV:GbCLA* vector after 2 weeks. (f, g) Disease symptoms and disease index of *TRV:00* and *TRV:GbCML45* seedlings at 21 dpi with *V. dahliae*. (h) Stem anatomy of *TRV:00* and *TRV:GbCML45* cotton plants shown in (f). (i) The relative biomass of *V. dahliae* in stems of *TRV:00* and *TRV:GbCML45* cotton plants. Data are means ± *SD* of three biological replicates (*n* = 3). Asterisks indicate statistically significant differences between *TRV:00* and *TRV:GbCML45* plants as determined by a Student's *t* test (*p < 0.05, **p < 0.01, ***p < 0.001).

To screen for candidate genes related to Verticillium wilt disease resistance in cotton, we analysed the root transcriptome data of two chromosome segment substitution 1 (CSSL1, resistant cultivar) and CSSL4 (susceptible cultivar) lines after being inoculated with *V. dahliae* (Zhang, Liu, et al., 2022). We found that the fragments per kilobase of transcript per million mapped reads (FPKM) values of the *Gbscaffold26027.2.0* gene (named *GbCML45* hereafter) were increased by about 10‐ and 30‐fold in the roots of resistant CSSL1 and susceptible CSSL4 cultivars, respectively, after their inoculation with *V. dahliae* for about 60 days in the disease nursery (Figure 1a,b). The phylogenetic tree analysis showed that GbCML45 has a conserved evolutionary relationship with those from other plant species that contain the EF‐hand domain (Figure S1a,b). Additionally, *Gb*/*GhCML45* was highly expressed in cotton roots, but expressed at low levels in stems and leaves in both the island (resistant cultivar Hai7124) and upland (*Gossypium hirsutum*, susceptible cultivar Jimian11) cotton plants (Figures S1c and S2a).

To confirm the up‐regulation of *CML45* expression after *V. dahliae* infection in our experimental system, we conducted a reverse transcription‐quantitative PCR (RT‐qPCR) assay to detect the expression levels of *GbCML45* and *GhCML45* in roots of the island and upland cotton plants in a time course after infection by a *V. dahliae* spore suspension (10^7^ spores/mL). There was an increase by approximately five‐ and three‐fold in the expression levels of *CML45* in the roots of both Hai7124 and Jimian11 plants at 0.25 and 3 days post‐inoculation (dpi), respectively (Figures 1c and S2b). To assess the role of *CML45* in *V. dahliae* resistance, we knocked down the expression of *CML45* in both Hai7124 and Jimian11 by using the virus‐induced gene silencing (VIGS) method in cotton. The expression levels of *GbCML45* and *GhCML45* were significantly lower in *TRV:GbCML45* and *TRV:GhCML45* plants than in the control *TRV:00* plants (Figures 1d and S2c). A marker gene, *chloroplastos alterados* (*CLA*), was silenced (*TRV:CLA*) to monitor the efficiency of VIGS at the same time, which indicated the proper action of the VIGS system through the albino phenotype of the newly grown true leaves (Figures 1e and S2d) (Gao et al., 2017). After their infection with *V. dahliae* for 21 days, there were more severe Verticillium wilt disease symptoms for both the *TRV:GbCML45* and *TRV:GhCML45* cotton plants (Figures 1f and S2e) and higher disease index values (Figures 1g and S2f) than *TRV:00* plants. Additionally, the *TRV:GbCML45* and *TRV:GhCML45* plants had more brown spots and lesion areas than *TRV:00* plants in their stem vascular tissues and more biomass of *V. dahliae* in stems (Figures 1h,i and S2g,h). These results indicated that the *V. dahliae*‐induced *GbCML45*/*GhCML45* positively regulated cotton resistance to *V. dahliae* infection.

### 2.2 GbCML45 interacts with GbCML50 and some TFs, and forms a homodimer

**FIGURE 2:**
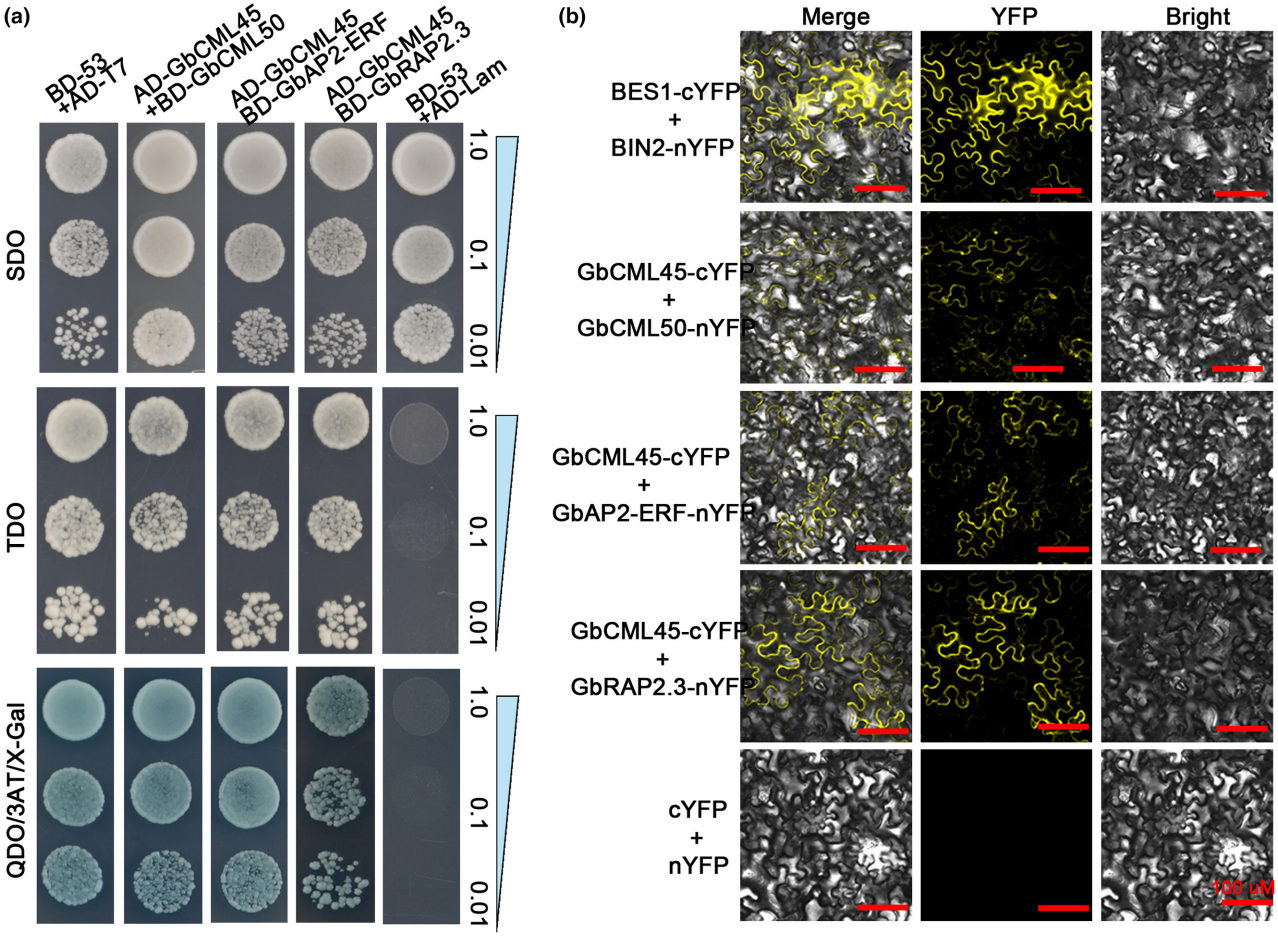
Interactions of GbCML45 with GbCML50, GbAP2‐ERF and GbRAP2.3 proteins. (a) Yeast two‐hybrid assays showing interaction between GbCML45 and GbCML50, GbAP2‐ERF and GbRAP2.3. Yeast growth with blue colonies indicate interaction. (b) Bimolecular fluorescence complementation assays showing interaction between GbCML45 and GbCML50, GbAP2‐ERF and GbRAP2.3 in *Nicotiana benthamiana* epidermal cells. GbCML45‐cYFP and GbCML50‐, or AP2‐ERF‐, or RAP2.3‐nYFP were co‐expressed in *N. benthamiana* leaves for the interaction assay. BES1‐cYFP and BIN2‐nYFP were used as positive control; bar = 100 μm. SDO (SD/−Trp); TDO (SD/−Ade/−His/−Trp); QDO (SD/−Trp/−Leu/−His/−Ade); BD‐53 + AD‐T7 (positive control); BD‐53 + AD‐Lam (negative control).

To identify some potential GbCML45‐interacting proteins in cotton, we constructed a yeast cDNA library using RNA from *V. dahliae*‐infected Hai7124 cotton roots and used GbCML45 as bait to perform a yeast two‐hybrid (Y2H) assay. Multiple putative GbCML45‐interactors were identified, and 56 positive yeast colonies were selected for sequencing and annotation. Three GbCML45‐interacting proteins were selected for further study: CML50, ERF and related to apetala (AP) 2.3 (RAP2.3) TFs (Table S1). Yeast colonies containing activation domain (AD)‐GbCML45 plus binding domain (BD)‐GbCML50, BD‐GbAP2‐ERF or BD‐GbRAP2.3 were able to grow in the presence of 3‐aminotriazole (3AT) and X‐Gal, verifying the interaction between GbCML45 and these identified proteins (Figure 2a). A bimolecular fluorescence complementation (BiFC) assay was conducted to further confirm the interaction of GbCML45 with GbCML50, GbAP2‐ERF and GbRAP2.3 in vivo. Yellow fluorescence was found in the cell membrane and nuclei when GbCML45‐cYFP and GbCML50‐nYFP were co‐expressed in *Nicotiana benthamiana* epidermal cells, while fluorescence was found only in the cell membrane when GbCML45‐cYFP was co‐expressed with GbAP2‐ERF‐nYFP or GbRAP2.3‐nYFP, strengthening the interaction of GbCML45 with GbCML50, GbAP2‐ERF and GbRAP2.3 in vivo (Figure 2b).

In addition, we found that GbCML45 could interact with itself and form a homodimer in the Y2H assay, while such homodimer formation was not detected in the case of GbCML50 in our Y2H assay (Figure S3a). These results were also verified by the BiFC assay where the homodimer formation of GbCML45 was observed as indicated by the yellow fluorescence in epidermal cells when GbCML45‐cYFP and GbCML45‐nYFP were co‐expressed. However, no fluorescence was detected in epidermal cells having GbCML50‐cYFP and GbCML50‐nYFP co‐expressed (Figure S3b).

### 2.3 Knockdown of *GbCML50* decreases Verticillium wilt resistance in cotton

**FIGURE 3:**
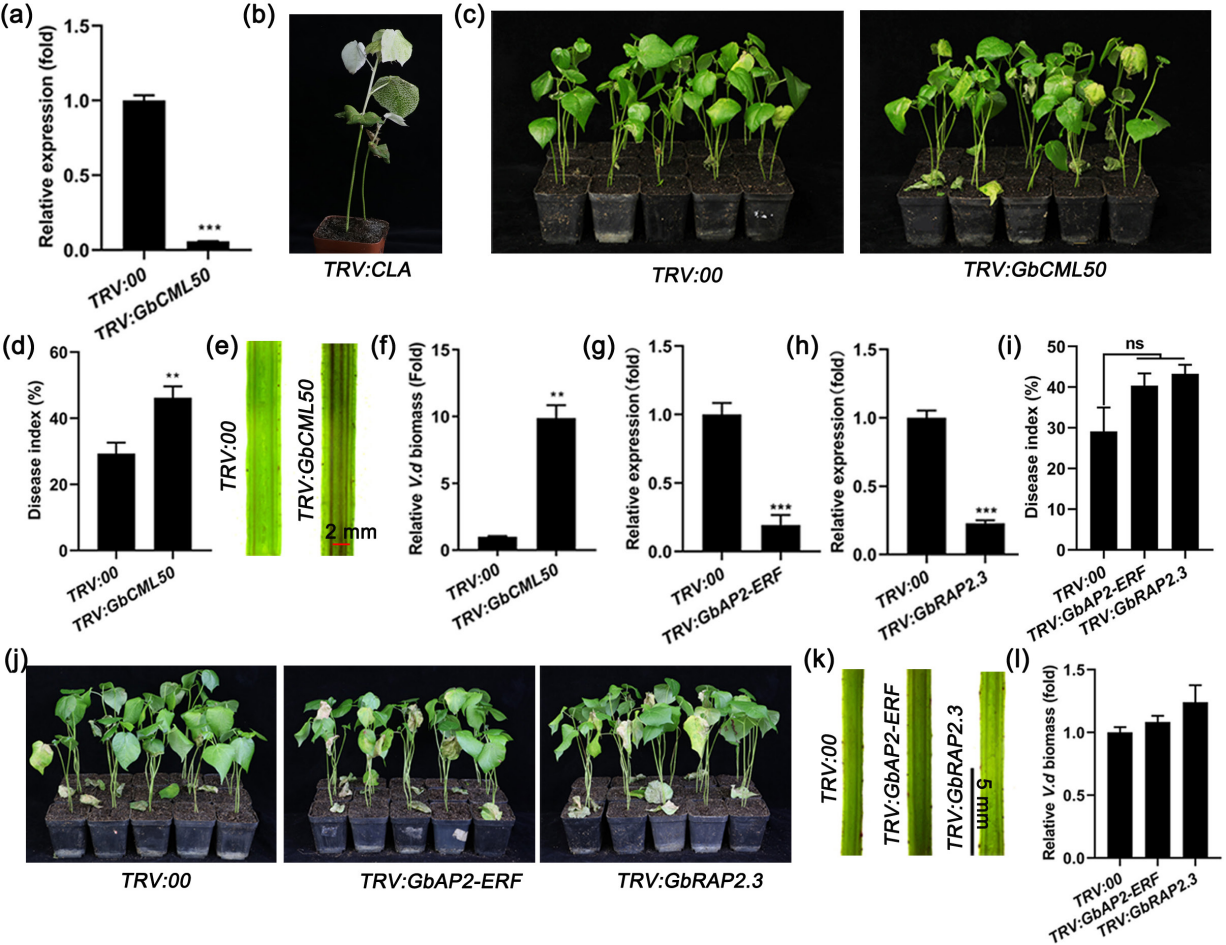
Silencing GbCML45‐interacting protein genes reduced resistance to *Verticillium dahliae* in cotton. (a) Relative transcript levels of *GbCML50* in roots of *TRV:GbCML50* cotton plants. (b) The albino phenotype of the cotton plants inoculated with *TRV:GbCLA* vector. (c, d) Disease symptoms and disease index of *TRV:00* and *TRV:GbCML50* seedlings at 21 days post‐inoculation (dpi) with *V. dahliae*. (e) Stem anatomy of *TRV:00* and *TRV:GbCML50* cotton plants shown in (c). (f) The relative biomass of *V. dahliae* in *TRV:00*, *TRV:GbCML50* cotton plants. (g, h) Relative transcript levels of *GbAP2‐ERF* and *GbRAP2.3* in roots of *TRV:GbAP2‐ERF* and *TRV:GbRAP2.3* cotton plants, respectively. (i, j) Disease symptoms and disease index of *TRV:00*, *TRV:GbAP2‐ERF* and *TRV:GbRAP2.3* seedlings at 21 dpi with *V. dahliae*. (k) Stem anatomy and *V. dahliae* recovery culture assay. (l) Relative biomass of *V. dahliae* in stems of *TRV:00*, *TRV:GbAP2‐ERF* and *TRV:GbRAP2.3* cotton plants. Data are means ± *SD* of three biological replicates (*n* = 3). Asterisks indicate statistically significant differences between *TRV:00* and *TRV:GbCML50* plants as determined by a Student's *t* test (ns, *p* > 0.05, **p < 0.01, ***p < 0.001).

Because *GbCML50* interacts with *GbCML45*, we asked if *GbCML50* is also involved in regulating the cotton response to *V. dahliae* infection. We knocked down *GbCML50* in the resistant Hai7124 cultivar using the VIGS method and assessed the responses of silenced plants to *V. dahliae* infection (Figure 3a,b). We found that *TRV:GbCML50* plants were more damaged by Verticillium wilt than *TRV:00* plants through observation of disease symptom and disease index (Figure 3c,d). Additionally, the *TRV:GbCML50* plants displayed deeper brown spots and more biomass of *V. dahliae* than *TRV:00* plants (Figure 3e,f). These results demonstrated that the knockdown of *GbCML50* increased cotton susceptibility to *V. dahliae* infection, and thus that *GbCML50* has a positive role in cotton Verticillium wilt resistance similar to its interacting partner *GbCML45*.

We also knocked down the *GbAP2‐ERF* and *GbRAP2.3* in similar manner to examine whether they function in cotton Verticillium wilt resistance (Figure 3g,h). After infection with *V. dahliae*, *TRV:GbAP2‐ERF* and *TRV:GbRAP2* plants exhibited more severe disease symptoms than *TRV:00* control plants; however, no significant differences in disease index value were recorded when comparing *TRV:GbAP2‐ERF* and *TRV:GbRAP2* plants with *TRV:00* plants (Figure 3i,j). Furthermore, the *TRV:GbAP2‐ERF* and *TRV:GbRAP2* cotton plants showed no obvious differences in terms of brown spots and biomass of *V. dahliae* in stems as compared with *TRV:00* plants (Figure 3k,l). On the basis of these findings, we subsequently focused on the mechanisms of *GbCML45* and *GbCML50* in regulating cotton resistance to Verticillium wilt.

### 2.4 Subcellular localization analysis and transcriptional activation assays of GbCML45 and 50

**FIGURE 4:**
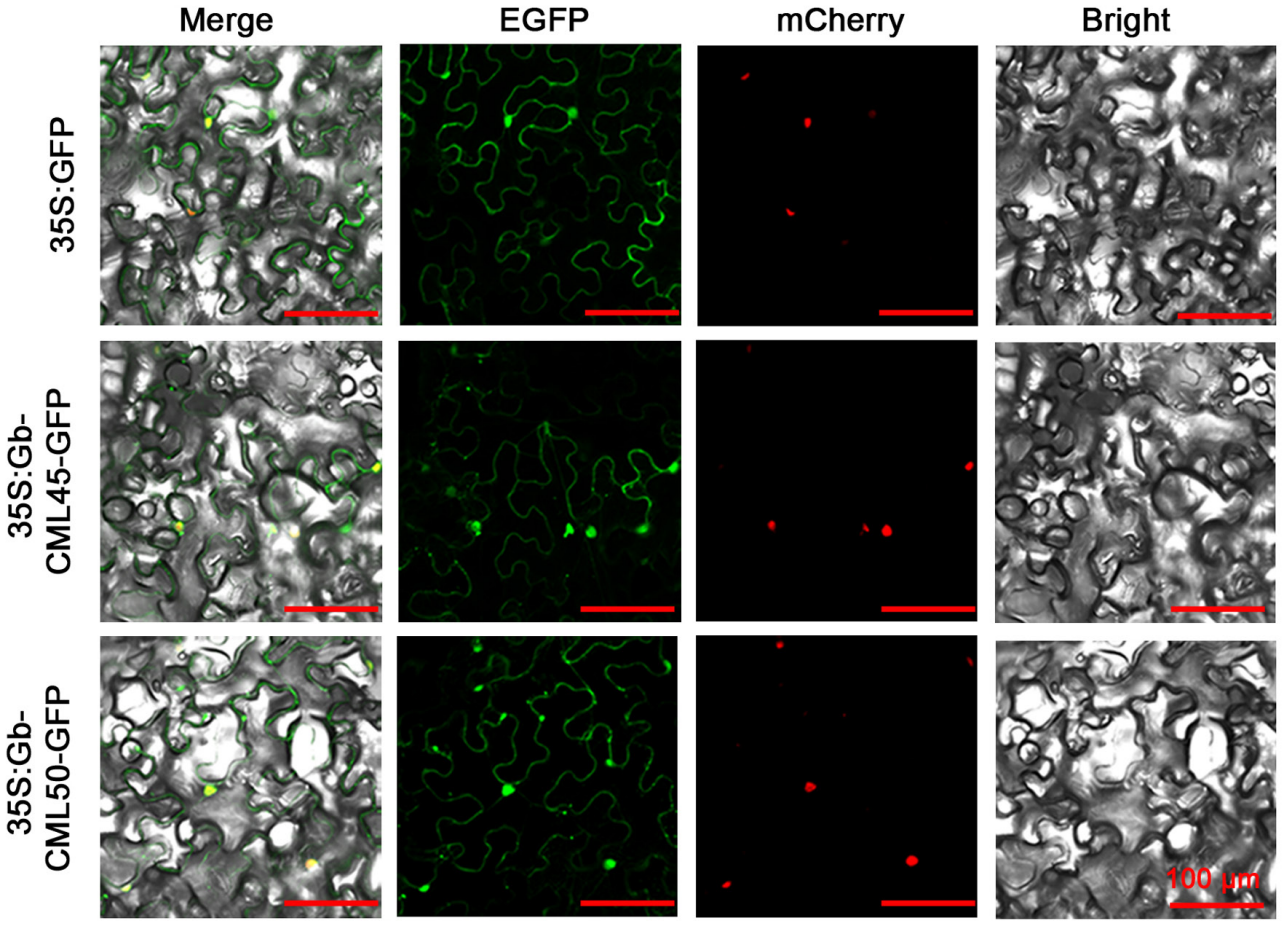
Subcellular localization and transcriptional activation of GbCML45 and GbCML50. Subcellular localization of GbCML45‐GFP and GbCML50‐GFP in leaves of *Nicotiana benthamiana*. The red fluorescence of H2B‐mCherry indicates the nucleus. Bar = 100 μm.

The subcellular localization of GbCML45 and GbCML50 in plant cells was investigated using an infiltration assay of the GFP‐fused constructs in *N. benthamiana* leaves. Green fluorescence detected in both cytoplasm and nuclei of epidermal cells supported the expression of the GbCML45‐GFP and GbCML50‐GFP constructs in these organelles (Figure 4).

### 2.5 Both *GbCML45* and *GbCML50* positively regulate Verticillium wilt resistance in *Arabidopsis* as their *Arabidopsis* homologues *AtCML46* and *AtCML49*

To examine the functions of *GbCML45* and *GbCML50* in regulating plant resistance to Verticillium wilt, we ectopically expressed each in *Arabidopsis* wild‐type (WT) plants (Figure S4a). Transgenic plants ectopically expressing *GbCML45* or *GbCML50* showed less severe disease symptoms, and lower disease index values and *V. dahliae* biomass than WT plants (Figure S4b–f). These results demonstrated the positive roles of *GbCML45* and *GbCML50* genes in regulating plant resistance to *V. dahliae* infection in the heterologous *Arabidopsis* system as well.

We also identified the closest orthologues of *GbCML45* and *GbCML50* in *Arabidopsis*, namely *AtCML46* (*AT5G39670*) and *AtCML49* (*AT3G10300*), and their corresponding loss‐of‐function mutants. The *Atcml46* and *Atcml49* mutants were then challenged with *V. dahliae* infection. At 14 dpi, more severe disease symptoms and higher disease index values were recorded for the *Atcml46* and *Atcml49* mutant plants than WT plants (Figure S4g,h). The *Atcml46* and *Atcml49* plants had more biomass of *V. dahliae* than WT plants (Figure S4i). These data suggest that *GbCML45* and *GbCML50* from cotton and their homologues from *Arabidopsis* have a conserved function, at least in terms of plant resistance to *V. dahliae* infection.

### 2.6 *V. dahliae* infection induces *GbCML45‐* and *GbCML50‐*mediated Ca^2+^ influx

**FIGURE 5:**
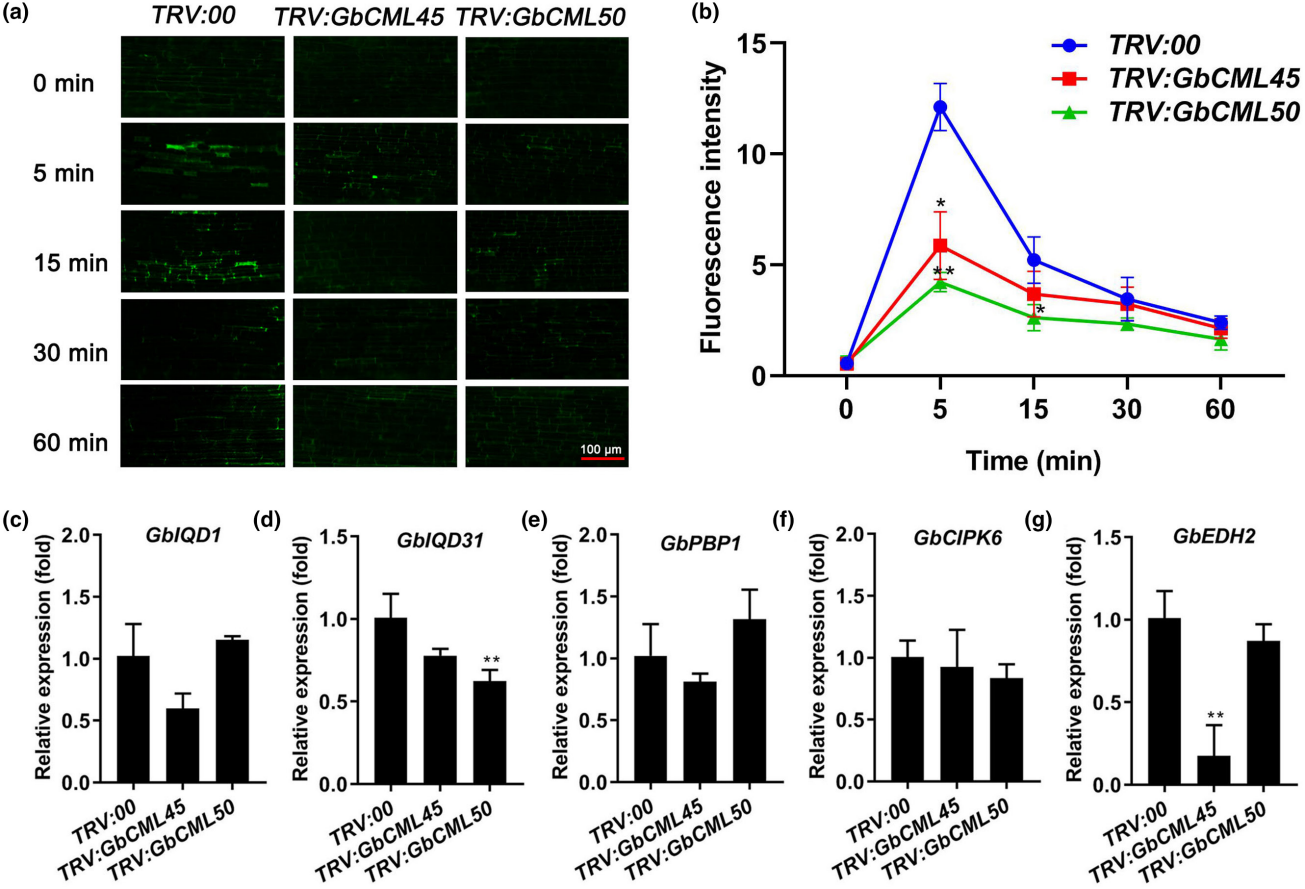
The Ca^2+^ levels and in expression levels of calcium signalling genes in cytosol of root cells in *TRV:00*, *TRV:GbCML45* and *TRV:GbCML50* cotton plants. (a) Fluorescence images of cotton root cells treated with Fluo‐4/AM at 0, 5, 15, 30 and 60 min after inoculation with *Verticillium dahliae*. The fluorescence signals were visualized by inverted fluorescence microscope. Scale bars = 100 μm. (b) Fluorescent intensity of *TRV:00*, *TRV:GbCML45* and *TRV:GbCML50* in cotton root cells. (c–g) Relative transcript levels of calcium signalling genes in *TRV:00*, *TRV:GbCML45* and *TRV:GbCML50* plants. These genes are calmodulin‐binding IQ‐domain protein genes *GbIQD1* (c) and *GbIQD31* (d), PINOID‐binding protein gene *GbPBP1* (e), CBL‐binding protein gene *GbCIPK6* (f), and Ca^2+^‐binding protein gene *GbEDH2* (g) (EPS15 homology domain protein). Data are presented as the mean ± *SD* (*n* = 3), and analysed using a two‐tailed Student's *t* test: *p < 0.05, **p < 0.01.

CMLs function as Ca^2+^ sensors in the earliest cellular responses to many abiotic/biotic stresses in plants (Zeng et al., 2023), implying that the roles of GbCML45 and GbCML50 in cotton plants might also be in charge of Ca^2+^‐mediated stress signal transduction for cotton Verticillium wilt resistance. To test this hypothesis, we performed a time course *V. dahliae* infection experiment to assess the change of Ca^2+^ levels in cotton root cytoplasm of *TRV:GbCML45* and *TRV:GbCML50* cotton plants. The Ca^2+^ fluorescence of cotton root cells was measured through Fluo‐4/AM indicator at 0, 5, 15, 30 and 60 min after the inoculation with *V. dahliae*. Our results showed that the fluorescence intensity in the root cells of *TRV:00* control plants significantly increased to the peak at 5 min after *V. dahliae* inoculation, and then decreased quickly (Figure 5a), indicating that *V. dahliae* infection of cotton roots induced Ca^2+^ influx into the cytosol. However, the fluorescence intensities in the cytosol of the root cells of *TRV:GbCML45* and *TRV:GbCML50* cotton plants were obviously weaker than in *TRV:00* plants at the 5 and 15 min time points (Figure 5a, b), suggesting that the *V. dahliae*‐induced Ca^2+^ influx into the cytosol was impaired by the silencing of either *GbCML45* or *GbCML50*. Consistently, the expression levels of several calcium signalling‐related genes, including the calmodulin‐binding *IQ‐domain protein* (*GbIQD1*) and (*GbIQD31*), *PINOID‐binding protein* (*GbPBP1*), *CBL‐interacting protein kinase* (*GbCIPK6*) and Ca^2+^‐binding protein EPS15 homology domain protein‐encoding *GbEHD2*, were down‐regulated in both *TRV:GbCML45* and *TRV:GbCML50* plants compared with *TRV:00* plants (Figure 5c–g).

### 2.7 *GbCML45* and *GbCML50* enhance cotton Verticillium wilt resistance dependent on SA and ET signalling pathways

**FIGURE 6:**
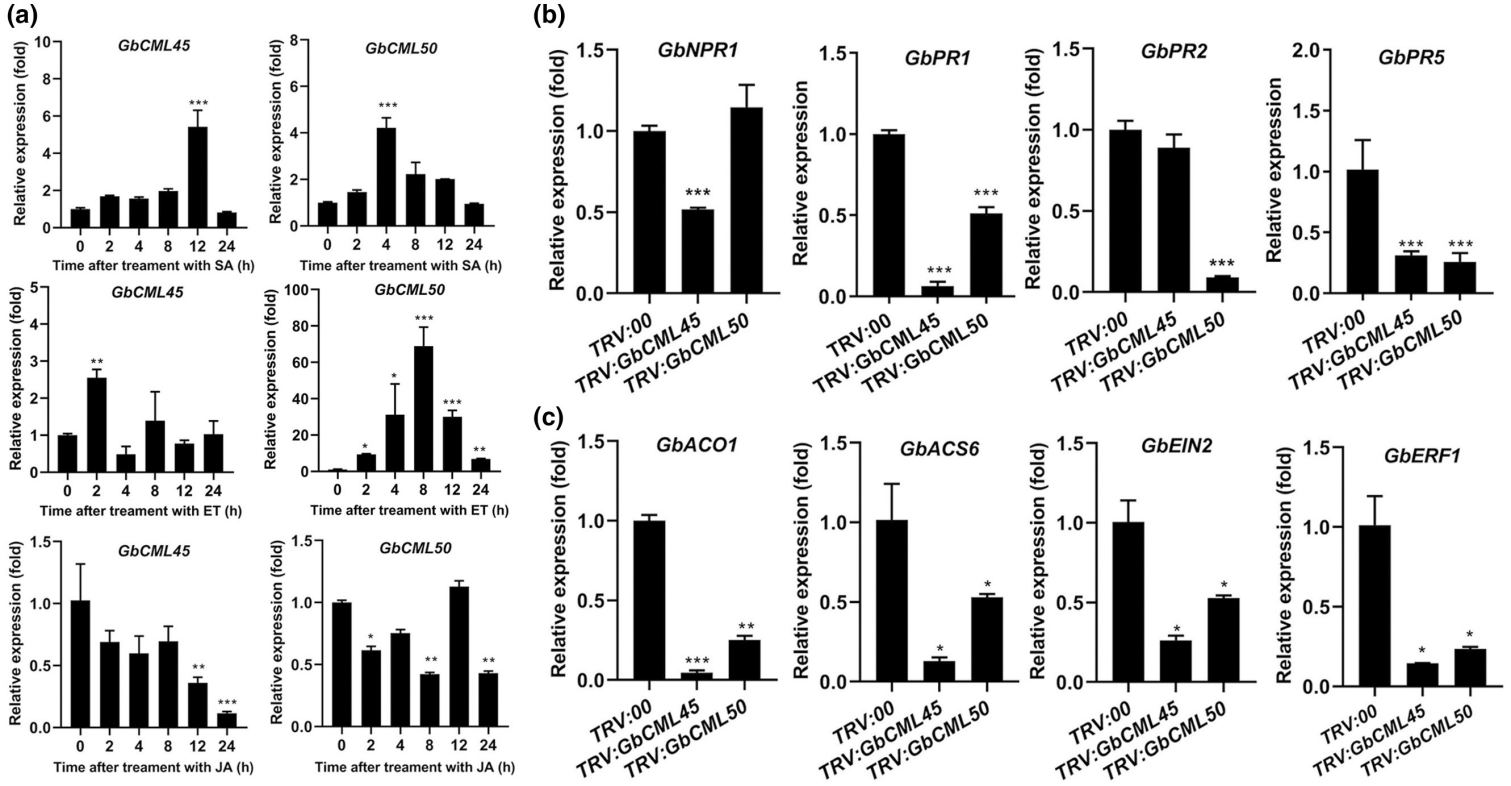
Relative transcript levels of *GbCML45* and *GbCML50* after treatment with hormones salicylic acid (SA), ethylene (ET) and methyl jasmonate (JA), and hormones or defence‐related genes in roots of *TRV:00*, *TRV:GbCML45* and *TRV:GbCML50* Hai7124 plants. (a) Relative transcript levels of *GbCML45* and *GbCML50* in roots of Hai7124 plants after treatment with 5 μM SA, ET or JA for 0, 2, 4, 8, 12 and 24 h. (b, c) Relative transcript levels of several defence‐related genes in SA and ET signal pathways, respectively, in roots of *TRV:00*, *TRV:GbCML45* and *TRV:GbCML50* Hai7124 plants. The expression value in *TRV:00* plants was normalized as 1. Data are means ± *SD* of three biological replicates (*n* = 3). Asterisks indicate statistically significant differences between *TRV:00*, *TRV:GbCML45* and *TRV:GbCML50* plants as determined by a Student's *t* test (*p < 0.05, **p < 0.01, ***p < 0.001).

Hormones such as SA, ET and methyl JA (MeJA) are associated with plant defence responses (Gupta et al., 2020; Li et al., 2019), and their crosstalk plays critical roles in Ca^2+^‐mediated immunity in plant (Heyer et al., 2022; Köster et al., 2022). Thus, we checked the expression levels of *GbCML45* and *GbCML50* following treatment with SA, ET and MeJA in cotton plants. Results revealed that the transcript levels of *GbCML45* and *GbCML50* were significantly increased by SA and ET treatments but decreased by MeJA treatment in cotton roots (Figure 6a), suggesting that the *CML45*‐ and *CML50*‐mediated cotton Verticillium wilt resistance might depend on the SA and ET signals rather than the JA signal. To further explore the role of *GbCML45*/*50* in SA and ET signalling pathways, we examined the expression of several defence‐related marker genes involved in these pathways in roots of *TRV:GbCML45*, *TRV:GbCML50* and *TRV:00* cotton plants. These marker genes included *GbNPR1* and *GbPR1*/*5*, involved in the SA signalling pathway, and *GbACS6*, *GbACO1*, *GbEIN2* and *GbERF1*, involved in the ET signalling pathway. The transcript levels of all these investigated marker genes were significantly down‐regulated in *TRV:GbCML45* and *TRV:GbCML50* cotton plants compared with the corresponding value in *TRV:00* control plants (Figure 6b,c). These findings collectively suggest that *GbCML45* and *GbCML50* might mediate cotton Verticillium wilt resistance downstream of the SA and ET signals and activate the expression of defence‐related genes involved in the SA and ET pathways.

### 2.8 *GbCML45* and *GbCML50* enhance Verticillium wilt resistance by inducing ROS and accumulation of NO, lignin and callose

**FIGURE 7:**
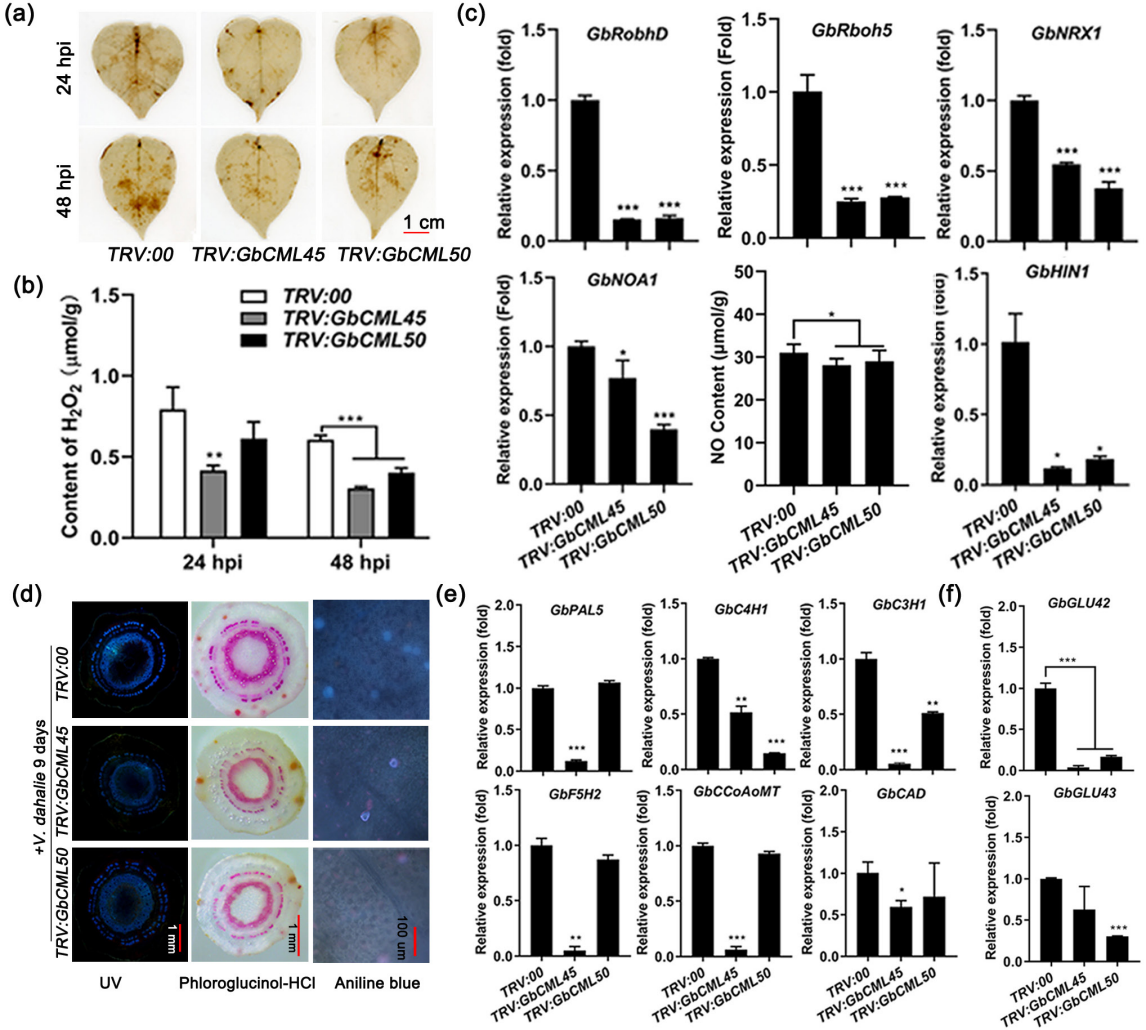
Accumulation of reactive oxygen species (ROS), lignin, callose and their production‐related genes expression in *GbCML45*‐ and *GbCML50*‐silenced Hai7124 cotton plants after inoculation with *Verticillium dahliae*. (a) 3,3′‐diaminobenzidine (DAB) staining of *GbCML45*‐ and *GbCML50*‐silenced cotton leaves to detect the O_2_^•−^ accumulation at 24 and 48 hours post‐inoculation (hpi) with *V. dahliae*. (b) Content of H_2_O_2_ in the *GbCML45*‐ and *GbCML50*‐silenced cotton roots at 24 and 48 hpi with *V. dahliae*. (c) Relative transcript levels of ROS production‐related genes and NO content in the *GbCML45*‐ and *GbCML50*‐silenced cotton roots at 48 hpi with *V. dahliae*. (d) Lignin spontaneous fluorescence under UV light and xylem phloroglucinol‐HCl staining in stem cross‐sections, and aniline blue staining of callose in leaf of *GbCML45*‐ and *GbCML50*‐silenced cotton plants. (e, f) Relative transcript levels of lignin and callose biosynthesis‐related genes in *GbCML45*‐ and *GbCML50*‐silenced cotton roots. Data are means ± *SD* of three biological replicates (*n* = 3). Asterisks indicate statistically significant differences between *TRV:00*, *TRV:GbCML45* and *TRV:GbCML50* plants as determined by a Student's *t* test (*p < 0.05, **p < 0.01, ***p < 0.001).

Increased intracellular Ca^2+^ and extensive interplay between Ca^2+^‐CaMs and ROS are common early cellular events to amplify plant immune signalling pathways in response to pathogen infection or elicitor treatment (Marcec et al., 2019; Zeng et al., 2023). To test the role of ROS in GbCML45/50‐mediated Verticillium wilt resistance, a 3,3′‐diaminobenzidine (DAB) staining assay was performed. Figure 7a showed that the ROS levels were lower in the leaves of the *TRV:GbCML45* and *TRV:GbCML50* plants than in those of *TRV:00* plants at 24 and 48 h post‐inoculation (hpi) with *V. dahliae*. The hydrogen peroxide (H_2_O_2_) contents were also significantly lower in the roots of *TRV:GbCML45* and *TRV:GbCML50* plants than in those of *TRV:00* plants, with the maximum decrease being observed at 24 and 48 hpi (Figure 7b). In addition to the reduced ROS accumulation, the expression levels of ROS production‐related genes *GbRbohD*, *GbRboh5* and *nucleoredoxin* (*GbNRX1*) were also reduced in the *TRV:GbCML45* and *TRV:GbCML50* cotton roots after 48 hpi with *V. dahliae* (Figure 7c). Similarly, the expression levels of NO synthesis‐related gene *NO associated 1* (*GbNOA1*) and the contents of NO were more reduced in *TRV:GbCML45* and *TRV:GbCML50* plants than in *TRV:00* plants (Figure 7c). These results indicated that *GbCML45* and *GbCML50* play an important role in regulating ROS and NO levels in plants cells to modulate cotton Verticillium wilt resistance at the early stage of *V. dahliae* infection.

Deposition of lignin and callose in the cell wall is important for plant defence against pathogen invasion as these components form a physical barrier to prevent the diffusion of pathogen‐produced toxins (Guo et al., 2016; Huang et al., 2021; Mbiza et al., 2022; Zhang et al., 2019). We asked if GbCML45 and GbCML50 play a role in lignin accumulation. We detected significantly lower lignin spontaneous fluorescence under UV light and lighter colour of xylem using phloroglucinol‐HCl staining in stem cross‐sections in *TRV:GbCML45* and *TRV:GbCML50* plants when compared with *TRV:00* plants after *V. dahliae* inoculation. Additionally, the deposition of callose was also reduced in leaves of *GbCML45*‐ and *GbCML50*‐silenced cotton plants after *V. dahliae* inoculation. These data indicated lower lignin and callose accumulation in *TRV:GbCML45* and *TRV:GbCML50* plants than in *TRV:00* plants in response to *Verticillium* infection (Figure 7d). Consistently, the expression of several key genes involved in the lignin biosynthesis pathway, including *GbPAL5*, *GbC4H1*, *GbC3H1*, *GbF5H2*, *GbCCoAOMT1* and *GbCAD9*, were significantly down‐regulated in the *TRV:GbCML45* and *TRV:GbCML50* plants when compared with the corresponding value in *TRV:00* plants (Figure 7e). The callose pathway genes *β‐1,3‐glucanase genes 42* and *43* (*GbGLU42* and *GbGLU43*), which are responsible for callose degradation and involved in Verticillium wilt resistance (Xu et al., 2016), were also significantly down‐regulated in the *TRV:GbCML50* and *TRV:GbCML45* plants (Figure 7f). These results indicated that *GbCML45* and *GbCML50* may positively regulate lignin and callose syntheses to enhance cotton Verticillium wilt resistance through improving physical barrier defence.

## 3 DISCUSSION

Cytosolic Ca^2+^ signalling plays a critical role in various aspects of plant growth, development and stress responses (Perochon et al., 2011). Perception of the Ca^2+^ signal through the binding of Ca^2+^ by the EF‐hand Ca^2+^‐motif of divergent forms of CaM/CML proteins can activate downstream components, triggering signal transduction (McCormack et al., 2005; Zhu et al., 2015). The expression patterns of *CML*s vary according to plant developmental stages, tissue types and environmental stimuli, indicating their specific roles in plant growth, development and responses to abiotic and biotic stresses (Cheval et al., 2013; Moore et al., 2011; Ranty et al., 2016). In this study, we found that *GbCML45* and its interacting partner *GbCML50* are important positive regulators of plant resistance to *V. dahliae* infection. This finding was supported by the fact that knockdown of either *GbCML45* or *GbCML50* increased cotton susceptibility to *V. dahliae* infection (Figures 1 and 3). Further analyses elucidated that GbCML45 and GbCML50 are both localized in the cytoplasm and nucleus and act in a Ca^2+^‐dependent manner to increase Verticillium wilt resistance (Figures 4 and 5). Earlier reports also showed that some CMLs, such as GhCML11 and GhCaM7 that are grouped in the same group III in the phylogenetic tree (Figure S5), function as positive regulators of upland cotton resistance to Verticillium wilt in a Ca^2+^‐dependent manner (Cheng et al., 2016; Zhang et al., 2023). However, GbCML45 and GbCML50 are located separately in groups IV and I, respectively, which are different from that of GhCML11 and GhCaM7, in the phylogenetic tree (Figure S5).

GhCML11 interacts with GhMYB108 TF and both act as positive regulators in cotton defence against *V. dahliae* infection (Cheng et al., 2016). In this study, we found that GbCML45 interacted with GbAP2‐ERF and GbRAP2.3 TFs in a Y2H assay, suggesting that different CMLs may have different interacting partners. Although silencing of GbAP2‐ERF and GbRAP2.3 caused an increase in disease symptom appearance on *TRV:GbAP2‐ERF* and *TRV:GbRAP2* plants (Figure 3j), the silenced plants did not show statistically significant differences, albeit an increased tendency, in disease index value when compared with *TRV:00* plants (Figure 3i,j). No obvious differences in phenotype of brown spots and the biomass of *V. dahliae* in stems, as compared with those in *TRV:00* plants, were found either (Figure 3k,l). These results suggest that the GbCML45‐interacting GbAP2‐ERF and GbRAP2 might have no or only a minor role in cotton resistance to *V. dahliae* infection. Taken together, CML proteins in cotton might interact and modulate the activities of some specific TFs, which in turn regulate the expression of downstream genes involved in cotton resistance to *V. dahliae* infection.

Biotic stresses have been reported to induce numerous cellular signalling events, including Ca^2+^ flux, transcriptional regulation, and the biosynthesis and signal transduction pathways of defence‐related hormones, such as SA, with the participation of CaMs/CMLs/CBPs (Zeng et al., 2023). In *Arabidopsis*, the knockout of pathogen‐inducible CaM‐binding protein CBP60g displayed an enhanced susceptibility to the bacterial pathogen *P. syringae* and reduced pathogen‐induced SA accumulation, demonstrating a positive regulatory role of CBP60g in SA production and pathogen resistance (Wang et al., 2009). On the other hand, constitutive insect herbivore resistance and elevated levels of SA have been observed in the *Arabidopsis* loss‐of‐function mutant defective in *signal responsive 1* (*AtSR1*), which encodes the calmodulin‐binding transcription activator 3 (CAMTA3) (Aldon et al., 2018; Galon et al., 2008; Qiu et al., 2012; Rahman et al., 2016). This CaM‐binding TF represses the expression of *enhanced disease susceptibility 1* (*EDS1*), a positive component of SA signalling (Du et al., 2009; Qiu et al., 2012). Additionally, CAMTA3 and its binding CaM negatively regulate SA accumulation and SA signalling to increase *Arabidopsis* susceptibility to *P. syringae* and the fungal pathogen *Botrytis cinerea* (Galon et al., 2008; Rahman et al., 2016). In our study, we observed that the expression of *GbCML45* and *GbCML50* was induced by exogenous SA (Figure 6a), indicating that SA acts upstream of *GbCML45* and *GbCML50* as a positive regulator to trigger their expression. This finding further suggests that SA may potentially be involved in *GbCML45*/*GbCML50*‐mediated cotton resistance to *V. dahliae* infection as a positive regulator. This premise was supported by the results that showed that the expression of *NPR1* and *PR* genes, involved in SA signalling‐related defence, were down‐regulated in both *GbCML45*‐silenced and *GbCML50*‐silenced plants (Figure 6b).

**FIGURE 8:**
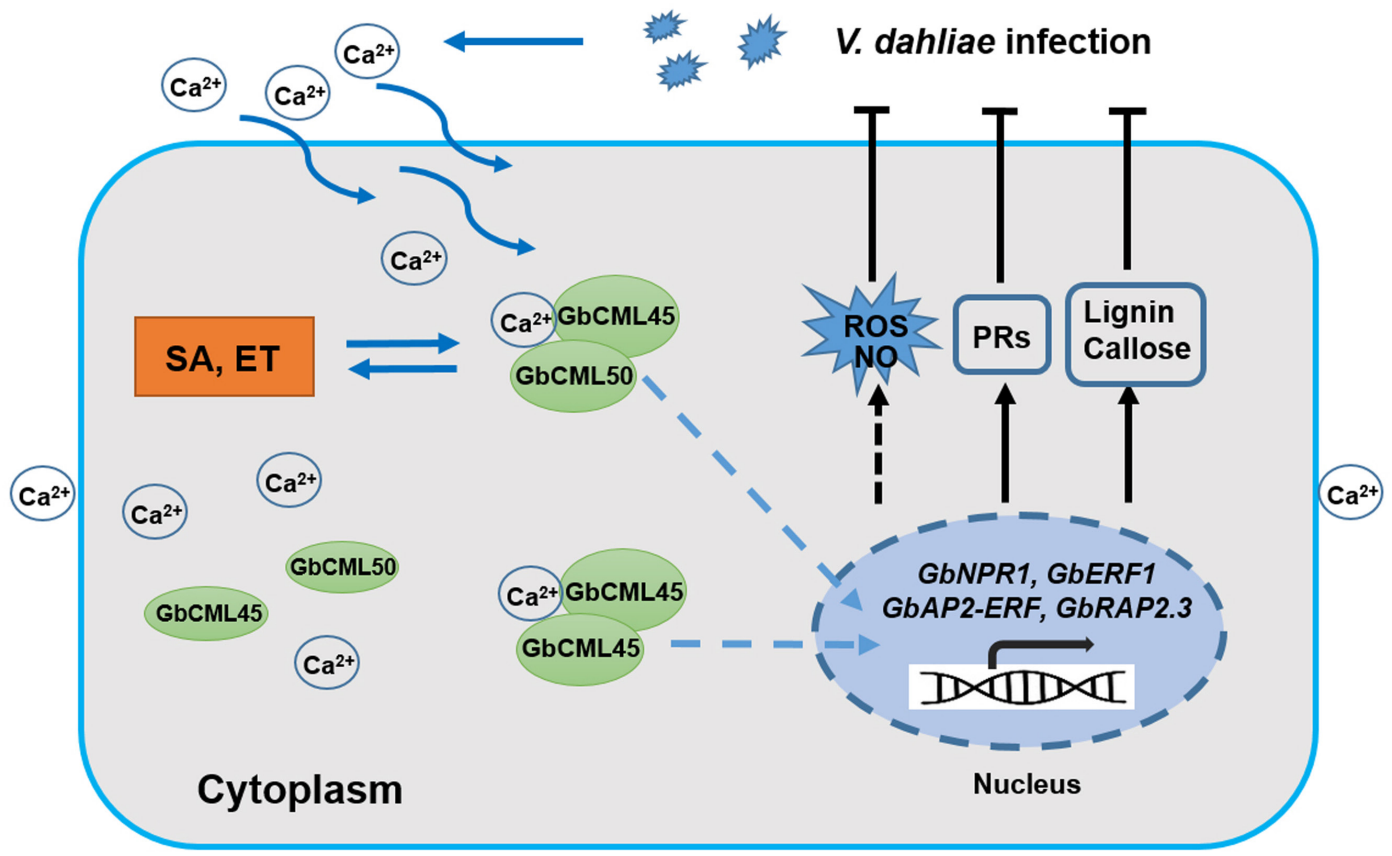
A proposed model for the role of *GbCML45* and *GbCML50* in regulating cotton resistance to *Verticillium dahliae* via interaction with salicylic acid (SA)‐ and ethylene (ET)‐signalling pathways and promoting reactive oxygen species (ROS) accumulation and lignin biosynthesis. *V. dahliae* infection induces the influx of Ca^2+^, which combines with the heterodimer GbCML45 and GbCML50 or homodimer GbCML45 and GbCML45, resulting in increasing ROS accumulation and NO production, and subsequently transmitting the defence signals into nucleus; simultaneously the signals are enhanced by SA and ET. In the nucleus, the expression of *GbAP2‐ERF*, *GbAP2.3*, *NPR1* and *ERF1* enhances expression of *PR* genes and lignin biosynthetic genes, resulting in increased cotton *V. dahliae* resistance. Black arrows indicate stimulation, black blunt bars indicate suppression, dotted arrows indicate uncertain stimulation.

Results of this study also indicated that the expression of *GbCML45* and *GbCML50* was induced by ET treatment (Figure 6a), suggesting that ET may also act upstream of *GbCML45*/*GbCML50*‐mediated cotton resistance to *V. dahliae* infection as a positive regulatory player similar to SA. This hypothesis was supported by two facts: (i) several signalling‐related (e.g., *GbEIN2* and *GbERF1*) genes were down‐regulated in both *GbCML45*‐silenced and *GbCML50*‐silenced cotton plants (Figure 6c) and (ii) several earlier studies also reported the positive role of ET signalling in cotton plant resistance to Verticillium wilt (Jia et al., 2022; Xiong et al., 2020; Yang et al., 2015). In addition, the ET biosynthesis‐related genes such as *GbACS6* and *GbACO1* were down‐regulated in *GbCML45*‐silenced and *GbCML50*‐silenced cotton plants (Figure 6c). These findings together suggest that *GbCML45* and *GbCML50* also regulate the expression of both the downstream genes of the ET signalling as well as the ET biosynthesis‐related genes in a feedback manner to trigger plant defence in response to Verticillium wilt (Figure 8). Roles of CaMs/CMLs/CBPs in ET‐modulated plant defence against pathogen infections have also been observed in other plant species. For example, ET‐mediated calcium‐dependent protein kinase 2 (NtCDPK2) was shown to promote biotic stress in *Nicotiana* plants with the tomato resistance gene *Cladosporium fulvum‐9* (*Cf* *‐9*) upon elicitation with the Avr9 peptide from the tomato pathogen *C. fulvum,* identified as an early gene‐for‐gene interaction race‐specific elicitor (Ludwig et al., 2005). Another example showed that the CaM‐binding TF AtSR1 enhanced plant defence against Pst DC3000 and ET‐induced senescence by directly promoting the expression of *ethylene insensitive 3* (*EIN3*) in *Arabidopsis* (Nie et al., 2012).

Both NO and ROS are important signalling molecules and act as downstream components in Ca^2+^ signalling in plant immunity processes. Previous investigations revealed that NO acts downstream of the Ca^2+^ signalling component in an early response to pathogen infection (Ali et al., 2007; Ma et al., 2008; Perochon et al., 2011). There are strong lines of evidence showing that NO and ROS accumulation is induced by overexpression of Ca^2+^‐CaM/CML proteins in the cytosol (Köster et al., 2022; Lamotte et al., 2004; Lecourieux et al., 2006; Ma et al., 2008; Ranty et al., 2016; Zeidler et al., 2004). For example, CaM1 promotes the production of NO, ROS and the expression of defence‐related genes, leading to resistance to bacterial pathogen *Xanthomonas campestris* pv. *vesicatoria* in leaves of pepper (Choi et al., 2009; Kim et al., 2014). In cotton, the GhCaM7‐enabled disease resistance to *V. dahliae* is related to the accumulation of NO and ROS (Zhang et al., 2023). This finding supports the positive correlation between reduced NO and ROS accumulation in *GbCML45*‐silenced and *GbCML50*‐silenced cotton plants and their susceptibility to *V. dahliae* infection (Figures 1d–i, 3a–f, and 7a–c). Collectively, GbCML45 and GbCML50 perceive the *V. dahliae*‐induced increase in intracellular Ca^2+^, which leads to increased NO and ROS production through up‐regulating the expression of related genes (Figure 7a–c). As a consequence, immunity responses are activated in cotton leading to Verticillium wilt resistance (Figure 8).

We found that lignin and callose accumulation was significantly reduced in stems of *GbCML45*‐ and *GbCML50*‐silenced cotton plants after *V. dahliae* inoculation (Figure 7d), which might contribute to the weakened resistance of the silenced plants to *V. dahliae* infection (Figure 7a), because the accumulation of lignin and callose is well known to contribute to enhanced resistance against *V. dahliae* in cotton through reinforcing cell wall structure (Huang et al., 2021; Mbiza et al., 2022; Xu et al., 2011). Similar results were also observed in other plant species in response to pathogen infection. For example, lignin deposition is rapidly induced after plant–pathogen interactions and enhances disease resistance to Pst DC3000 (*AvrRpm1*) in *Arabidopsis* (Kim et al., 2020; Lee et al., 2019). PAMPs (e.g., flagellin and chitin) induce cytosolic Ca^2+^ influx and callose deposition in the elongation zone of primary roots, supporting a correlation between Ca^2+^ level and callose accumulation (Dubiella et al., 2013). *Arabidopsis AtCML9*‐overexpressing lines exhibit reduced susceptibility to *P. syringae* infection by deposition of callose and modification of callose biosynthesis‐related gene expression (Leba et al., 2012). Consistently, the expression of *AtCML41* was up‐regulated by flagellin treatment, which facilitates callose deposition at plasmodesmata, and enhances plant defence against *P. syringae* (Xu et al., 2017). Our results suggested that lignin and callose deposition induced by *GbCML45* and *GbCML50* also plays a critical contribution to Verticillium wilt resistance in cotton. The fast and efficient cotton transformation method using shoot apical meristem cell‐mediated transformation system (SAMT; Ge et al., 2023) could be used to create cotton materials overexpressing *GbCML45* and *GbCML50* to enhance the disease resistance of cotton varieties.

In conclusion, our results illustrated that *V. dahliae* infection induces the influx of Ca^2+^ into cytosol, which activates GbCML45 and GbCML50 and the related TFs, resulting in increased ROS and NO accumulation, up‐regulated expression of genes involved in SA and ET signalling pathways like *NPR1* and *ERF1*, up‐regulated expression of *PR* genes and accumulation of lignin and callose, leading to enhanced cotton *V. dahliae* resistance (Figure 8). Our results provide new insights into the Ca^2+^‐CML signalling‐mediated cotton Verticillium wilt resistance, with the example of GbCML45 and GbCML50, which eventually will lead to new targets for molecular breeding cultivars with improved resistance to Verticillium wilt in cotton.

## 4 EXPERIMENTAL PROCEDURES

### 4.1 Plant materials and growth conditions

In this study, the island cotton cultivar Hai7124 and the upland cotton cultivar Jimian11 were used. For germination, seeds of cotton were first imbibed in water for 8 h at 28°C in the dark in an incubator. Three days later, the germinating seedlings were transferred to soil in pots under an 8 h light 22°C/16 h dark 25°C photoperiod/temperature cycle for 10 days. These 10‐day‐old seedlings were then used for VIGS infiltration. For *Arabidopsis*, the Columbia‐0 (Col‐0) ecotype was used as the WT, and the ectopically expressing *GbCML45* or *GbCML50 Arabidopsis* transgenic lines were obtained using the vector of pCAMBIA2300‐GFP. Seeds of Col‐0 background *Atcml46* (SALK_127471C) and *Atcml49* (SALK_035905C) were obtained from Salk Institute Genomic Analysis Laboratory (http://signal.salk.edu/).

### 4.2 Phylogenetic analysis and conserved domain architecture construction

MUSCLE in MEGA 7 was used to align GbCML45, GbCML50 and other related protein sequences (Kumar et al., 2016). To construct an unrooted phylogenetic tree, the neighbour‐joining method and the Jones–Taylor–Thornton model were used through 1000 bootstrap replicates. To visualize the phylogenetic tree, Evolview (https://www.evolgenius.info) was used (Balakrishnan et al., 2019). To determine the gene conserved domain, Gene Structure Display Server (GSDS: http://gsds.cbi.pku.edu.cn/) was performed based on the corresponding genomic coding sequences (Li et al., 2014).

### 4.3 Cultivation of *V. dahliae*, gene cloning, VIGS vector construction and western blot analysis

The *V. dahliae* strain V991 was used in our experiment, which was highly aggressive, defoliating and stored in 30% glycerol at −80°C. The activation and infection processes were same as previously described (Gao et al., 2013). To clone *GbCML45* and *GbCML50* genes, the respective coding sequences were obtained from the cotton database by sequence BLAST (https://cottonfgd.org/sequenceserver/). Primers were designed using Premier 6.0 (http://www.premierbiosoft.com/) to amplify the *GbCML45* and *GbCML50* sequences from Hai7124. The detailed processes of plasmid construction, gene transformation and expression and detection of OE‐GbCML45‐GFP protein were described previously (Yi et al., 2023).

### 4.4 VIGS transient expression and pathogen infection assays

The VIGS transient expression assay was the same as previously described (Gao et al., 2011). All VIGS‐infiltrated seedlings, including *TRV:GbCLA, TRV:GbCML45*, *TRV:GbCML50, TRV:GbCLA* Hai7124 and *TRV:00*, were grown for 2 weeks. The detailed process of pathogen infection, proportion of diseased plants and calculation of the disease index were as previously described (Yan et al., 2016; Yi et al., 2023).

### 4.5 Section anatomy in stems and fungal quantification

To observe sections of cotton plants, stems were cut into cross‐sections manually and a stereomicroscope (Olympus) was used for observation and photographing. Quantification of *V. dahliae* biomass was performed in accordance with previously described methods (Jia et al., 2022).

### 4.6 RNA isolation, RT‐qPCR and analyses and observation of subcellular localization

Total RNA was extracted from cotton leaves, stems and roots using the FastPure Universal Plant Total RNA isolation kit (Nanjing Vazyme Biotech Co., Ltd). The first‐strand cDNA, qPCR assay, and relative gene expression level procedures were the same as previously described (Rieu & Powers, 2009; Yi et al., 2023) with the cotton *UBQ7* gene (*GhUB7*, accession: DQ116441) used as the internal control. Gene‐specific primers used for RT‐qPCR are shown in Table S2. To observe subcellular localization of GbCML45, GbCML50, GbAP2‐ERF and GbRAP2.3, the genes were amplified and fused in vectors p*35S‐GFP:GbCML45*, p*35S‐GFP:GbCML50*, p*35S‐GFP:GbAP2‐ERF* and p*35S‐GFP:GbAP2.3*, which were subsequently transformed into *Agrobacterium tumefaciens* GV3101. Details of the process and observation were as described previously (Yi et al., 2023).

### 4.7 Y2H and BiFC assays

The yeast library construction and Y2H assays were conducted as described in the manufacturer's instructions of Match‐maker Gold Yeast Two‐Hybrid System (Clontech). The AD‐GbCML45 and BD‐GbCML50, BD‐GbAP2‐ERF and BD‐GbRAP2.3 were constructed to test the interactions between them. Mating between yeast strains that contained AD or BD constructs was performed and screened on amino acid‐deficient medium. After SD/−Trp/−Leu (DDO) screening, positive colonies were plated onto SD/−Trp/−Leu/−His/−Ade (QDO) supplemented with X‐Gal for further validation. Media were supplemented with 10 mM 3‐aminotriazole (3‐AT) to prevent auto‐activation.

For BiFC assays, the coding sequence of *GbCML45* was cloned into pXY104 (cYFP) vector, while *GbCML50*, *GbAP2‐ERF* and *GbRAP2.3* were cloned into pXY106 (nYFP). The generated constructs of GbCML45‐cYFP, GbCML50‐nYFP, GbAP2‐ERF‐nYFP and GbRAP2.3‐nYFP were individually transformed into *A. tumefaciens* GV3101 and were then co‐expressed GbCML45‐cYFP with GbCML50‐nYFP, GbAP2‐ERF‐nYFP and GbRAP2.3‐nYFP, respectively, in *N. benthamiana* leaves through *Agrobacterium* injection. BES1‐cYFP and BIN2‐nYFP were co‐expressed as the positive control (Gao et al., 2020). Three days after inoculation, yellow fluorescence signals were detected with a confocal microscope (LSM710; Zeiss).

### 4.8 Transcriptional activation assay

The coding sequences of *GbCML45* and *GbCML50*, *GbAP2‐ERF* and *GbRAP2.3* were fused with the pGBKT7 vector to generate BD‐GbCML45, BD‐CML50, BD‐AP2‐ERF and BD‐RAP2.3. The plasmid pGBKT7‐53 was used as positive control. Each vector was transformed into the Y2H gold yeast strain and plated on SD/−Trp (SDO) medium for positive selection. Dilutions of yeast clones were then plated onto SD/−Trp/−His/−Ade (TDO) and TDO/X‐Gal media for subsequent transcriptional activation assays. Images were taken after incubation on the medium for 3 days.

### 4.9 Cytosolic Ca^2+^ signal detection

Cytosolic Ca^2+^ staining and observation was performed as described by a previous study (Cheng et al., 2016). The fluorescence intensity of root cells was determined using ImageJ software.

### 4.10 Hormone application

Chemicals of SA, ethrel (donor of ET) and MeJA were dissolved in ethanol to make 10 mM stock solutions. To prepare the working solutions, distilled water was added to dilute stock solutions to 10 μM, and same amount of ethanol was also added in distilled water as the control (mock). Three or four true leaves cotton seedlings were used for SA, ET and MeJA application by foliar spraying or soil drenching. Leaf and root samples were harvested and put into storage bags and frozen in liquid nitrogen and stored at −80°C for further analysis. Three biological replicates were used for each treatment.

### 4.11 Histochemical assay and H_2_O_2_ and NO measurement

Content of H_2_O_2_ was examined by staining with DAB, which was determined using the method previously described (Wang et al., 2019). H_2_O_2_ and NO contents were determined by using detection kits (Solarbio), detailed operation steps were performed as in the manual instructions. All analyses had three biological replicates.

### 4.12 Observation of lignin synthesis and callose accumulation

To observe cotton seedlings of *GbCML45*‐ and *GbCML50*‐silenced plants, stem sections of these plants were first incubated in 10% phloroglucinol solution for 2 min. Then, the samples were soaked in concentrated HCl for about 5 min and the staining signals observed through a microscope. To observe callose, the true leaves of the control and *GbCML45*‐ and *GbCML50*‐silenced cotton were harvested. To remove chlorophyll, the leaves were fixed in fixative solution (ethanol:acetic acid 3:1) for 2 h, and next soaked in 70% and 50% ethanol for 2 h, then finally incubated in distilled water overnight. To make samples transparent, after rinsing with water, the leaves were soaked in 10% NaOH for 1 h. The callose content was observed under a fluorescence microscope with UV excitation light after being kept in darkness in 0.01% aniline blue for 3 h (Huang et al., 2021). All experiments were repeated at least three times.

## CONFLICT OF INTEREST STATEMENT

The authors declare that no competing interests exist.

## Supporting information


**FIGURE S1.** Phylogenetic tree and functional domain of calmodulin‐like protein CML45 in different plant species, and gene expression patterns of *GbCML45* in different tissues of Hai7124 cotton plants. (a,b) The phylogenetic tree and conserved domain of CML45 in different plant species. The phylogenetic tree was constructed by the neighbour‐joining (NJ) method, with 1000 bootstrap replicates. The colour boxes indicate different conserved domain. *Gbar* (*Gossypium barbadense*); *Gohir* (*Gossypium hirsutum*); *Ga* (*Gossypium arboretum*); *Gorai* (*Gossypium raimondii*); *Glyma* (*Glycine max*); *VIT* (*Vitis vinifera*); *Traes* (*Triticum aestivum*). (c) The expression patterns of *GbCML45* in leaves, stems and roots of Hai7124 cotton plants. Data are presented as the mean ± *SD* (*n* = 3) and analysed using a two‐tailed Student’s *t*test: *p < 0.05, ***p < 0.001.

**FIGURE S2.** Silencing *GhCML45* in upland cotton (*Gossypium hirsutum*) plants reduced *Verticillium dahliae* resistance. (a) The expression patterns of *GhCML45* in leaves, stems and roots of Hai7124 cotton plants. (b) The transcript levels of *GhCML45* in the roots of upland cotton (Jimian11) after 0, 0.25, 0.5, 1, 3, 5, 9 and 12 days post‐inoculation (dpi) with *V. dahliae* infection. (c) The relative transcript levels of *GhCML45* in roots of *TRV:GhCML45* plants. (d) Albino phenotype of the *TRV:GhCLA* plants. (e) Disease symptoms of *TRV:00* and *TRV:GhCML45* plants at 21 dpi with *V. dahliae*. (f) Disease index of *TRV:00* and *TRV:GhCML45* seedlings. (g) Section anatomy of stems from (e). (h) Percentage of lesion area of cotton stem section from (g). Data are means ± *SD* of three biological replicates (*n* ≥ 25) and analysed using a two‐tailed Student’s *t*test: **p < 0.01, ***p < 0.001.

**FIGURE S3.** GbCML45 can interact with itself to form a dimer. (a) Yeast two‐hybrid assays showing interaction between GbCML45 and itself, but not happened in GbCML50 proteins. Yeast growth indicate interaction. (b) Bimolecular fluorescence complementation assays showing interaction between GbCML45 and itself in *Nicotiana benthamiana* epidermal cells. BES1‐cYFP and BIN2‐nYFP were used as positive control; bar = 100 μm. SDO (SD/−Trp); QDO (SD/−Trp/−Leu/−His/−Ade); BD‐53 + AD‐T7 (positive control); BD‐53 + AD‐Lam (negative control).

**FIGURE S4.** *GbCML45* and *GbCML50* overexpression *Arabidopsis thaliana* plants enhanced resistance to *Verticillium dahliae* infection, while reduced in the *Atcml45* and *Atcml 50 A. thaliana* mutant plants. (a) Western blotting was used to detect the accumulation of GbCML45‐GFP and GbCML50‐GFP in different transgenic lines. Primary antibody of anti‐GFP mouse monoclonal antibody and the secondary antibody of horseradish peroxidase‐conjugated goat anti‐mouse IgG were used. (b) Disease symptoms of wild‐type (WT) and transgenic *Arabidopsis* plants ectopically expressing *GbCML45* and *GbCML50* plants at 15 days post‐inoculation (dpi) with *V. dahliae*. Photographs were taken at 15 dpi, and for each line at least 24 plants were observed. (c,d) Disease indices of wild‐type and *GbCML45*‐ and *GbCML50‐*overexpressing lines shown in (b). (e,f) Relative biomass of *V. dahliae* in *GbCML45*‐ and *GbCML50‐*overexpressing lines. (g) Disease symptoms of *Atcml46* (*GbCML45* orthologues) and *Atcml49* (*GbCML50* orthologues) mutant plants at 14 dpi with *V. dahliae*. (h) Disease index of *Atcml46* and *Atcml49* seedlings at 14 dpi with *V. dahliae*. (i) Relative biomass of *V. dahliae* in *Atcml46* and *Atcml49* seedlings. Data are means ± *SD* of three biological replicates (*n* = 3; ≥24 independent plants/biological replicate). Data was analysed using a two‐tailed Student’s *t*test: **p < 0.01, ***p < 0.001.

**FIGURE S5.** Phylogenetic analysis of CMLs in cotton and *Arabidopsis*. A phylogenetic tree of CML proteins from *Gossypium barbadense* and *Arabidopsis thaliana*. The full‐length amino acid sequences of the CML proteins were aligned using ClustalX in MEGA 7.0. The unrooted tree was generated by the neighbour‐joining method (*n* = 1000 bootstraps). Cotton CMLs are coloured with red arrows and the *Arabidopsis* CMLs are in blue arrows. Different coloured solid circles indicate genes in different groups of *Gossypium* species or *Arabidopsis*.

**Table S1.** Screening yeast library of cotton root infected by *Verticillium dahliae*.

**Table S2.** (a) List of primers used in reverse transcription‐quantitative PCR analysis. (b) List of primers used in vector construction.

**Table S3.** Major genes used in this study.

## Data Availability

Data that support the findings of this study are available from the corresponding author upon reasonable request. The major genes/proteins sequence mentioned in this study is shown in Table S3 and can be obtained from the online cotton database (https://cottonfgd.org/sequenceserver/).
