# Supplementary material for: Positive roles of the Ca2+ sensors GbCML45 and GbCML50 in improving cotton Verticillium wilt resistance
Source: Mol Plant Pathol. 2024 Jun 3;25(6):e13483. doi: 10.1111/mpp.13483 (PMC11146148; doi:10.1111/mpp.13483)
Supplement: Supplementary file 1 — FIGURE S1. Phylogenetic tree and functional domain of calmodulin‐like protein CML45 in different plant species, and gene expression patterns of GbCML45 in different tissues of Hai7124 cotton plants. (a,b) The phylogenetic tree and conserved domain of CML45 in different plant species. The phylogenetic tree was constructed by the neighbour‐joining (NJ) method, with 1000 bootstrap replicates. The colour boxes indicate different conserved domain. Gbar (Gossypium barbadense); Gohir (Gossypium hirsutum); Ga (Gossypium arboretum); Gorai (Gossypium raimondii); Glyma (Glycine max); VIT (Vitis vinifera); Traes (Triticum aestivum). (c) The expression patterns of GbCML45 in leaves, stems and roots of Hai7124 cotton plants. Data are presented as the mean ± SD (n = 3) and analysed using a two‐tailed Student’s ttest: *p < 0.05, ***p < 0.001. [file MPP-25-e13483-s003.docx]

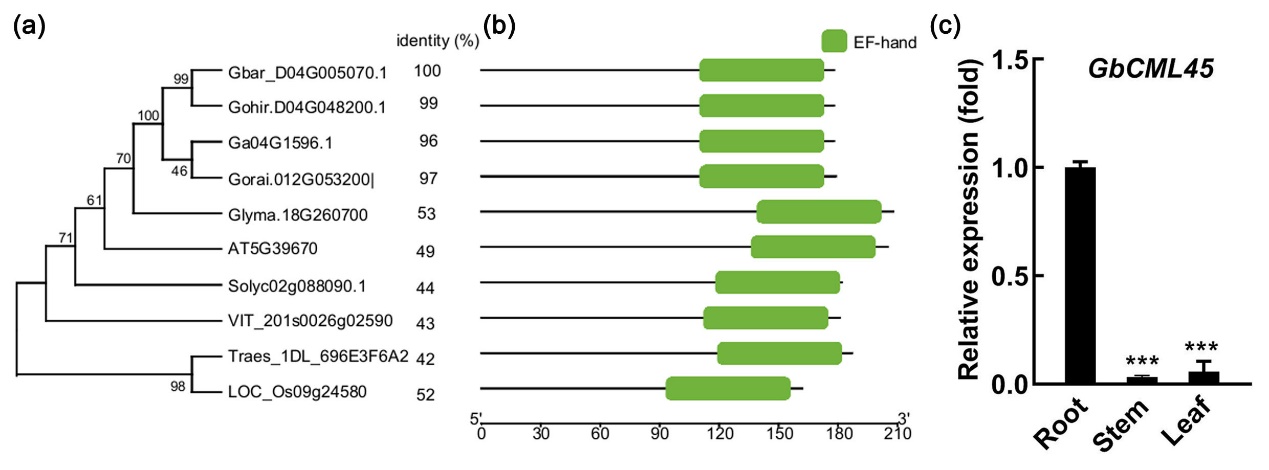


**Figure S1.** Phylogenetic tree and functional domain of Calmodulin-Like protein CML45 in different plant species, and gene expression patterns of *GbCML45* in different tissues of Hai7124 cotton plants. (a-b) The phylogenetic tree and conserved domain of CML45 in different plant species. The phylogenetic tree was constructed by the Neighbor-Joining (NJ) method, with 1,000 bootstrap replicates. The color boxes indicate different conserved domain. *Gbar*, (*Gossypium barbadense*); *Gohir*, (*Gossypium hirsutum*); *Ga*, (*Gossypium* *arboretum*); *Gorai*, (*Gossypium raimondii*); *Glyma*, (*Glycine max*); *VIT*, (*Vitis vinifera*); *Traes*, (*Triticum aestivum*); (c) The expression patterns of *GbCML45* in leaves, stems and roots of Hai7124 cotton plants. Data are presented as the mean ± standard deviation (SD) (*n* = 3), and analyzed using a two-tailed Student’s *t*-test: *, *P* < 0.05; ***, *P* < 0.001.
