# Supplementary material for: Positive roles of the Ca2+ sensors GbCML45 and GbCML50 in improving cotton Verticillium wilt resistance
Source: Mol Plant Pathol. 2024 Jun 3;25(6):e13483. doi: 10.1111/mpp.13483 (PMC11146148; doi:10.1111/mpp.13483)
Supplement: Supplementary file 2 — FIGURE S2. Silencing GhCML45 in upland cotton (Gossypium hirsutum) plants reduced Verticillium dahliae resistance. (a) The expression patterns of GhCML45 in leaves, stems and roots of Hai7124 cotton plants. (b) The transcript levels of GhCML45 in the roots of upland cotton (Jimian11) after 0, 0.25, 0.5, 1, 3, 5, 9 and 12 days post‐inoculation (dpi) with V. dahliae infection. (c) The relative transcript levels of GhCML45 in roots of TRV:GhCML45 plants. (d) Albino phenotype of the TRV:GhCLA plants. (e) Disease symptoms of TRV:00 and TRV:GhCML45 plants at 21 dpi with V. dahliae. (f) Disease index of TRV:00 and TRV:GhCML45 seedlings. (g) Section anatomy of stems from (e). (h) Percentage of lesion area of cotton stem section from (g). Data are means ± SD of three biological replicates (n ≥ 25) and analysed using a two‐tailed Student’s ttest: **p < 0.01, ***p < 0.001. [file MPP-25-e13483-s002.docx]

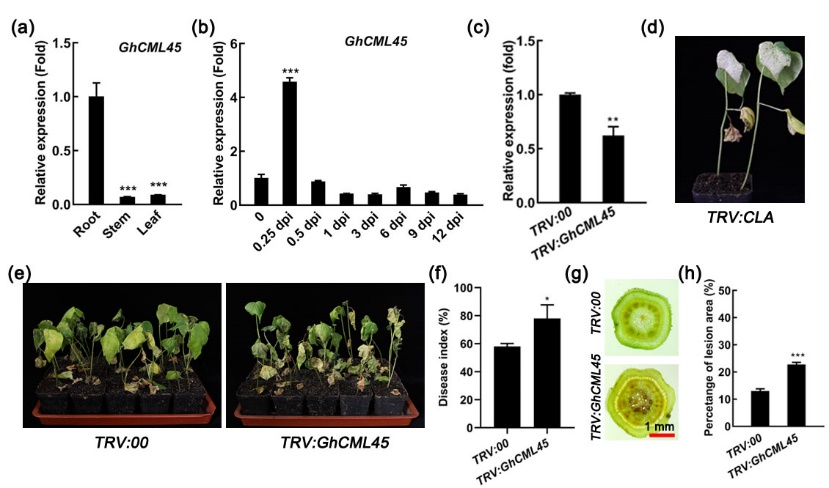


**Figure S2.** Silencing *GhCML45* in upland cotton (*Gossypium hirsutum*) plants reduced *Verticillium dahliae* resistance. (a) The expression patterns of *GhCML45* in leaves, stems and roots of Hai7124 cotton plants. (b) The transcript levels of *GhCML45* in the roots of upland cotton (Jimian11) after 0, 0.25, 0.5, 1, 3, 5, 9 and 12 dpi with *V. dahliae* infection. (c) The relative transcript levels of *GhCML45* in roots of *TRV:GhCML45* plants. (d) Albino phenotype of the *TRV:GhCLA* plants. (e) Disease symptoms of *TRV:00* and *TRV:GhCML45* plants at 21 dpi with *V. dahliae*. (f) Disease index of *TRV:00* and *TRV:GhCML45* seedlings. (g) Section anatomy of stems from (e). (h) Percentage of lesion area of cotton stem section from (g). Data are means ± SD of three biological replicates (*n* ≥ 25), and analyzed using a two-tailed Student’s *t*-test: **, *P* < 0.01; ***, *P* < 0.001. dpi, day-post-inoculation.
