# Supplementary material for: Positive roles of the Ca2+ sensors GbCML45 and GbCML50 in improving cotton Verticillium wilt resistance
Source: Mol Plant Pathol. 2024 Jun 3;25(6):e13483. doi: 10.1111/mpp.13483 (PMC11146148; doi:10.1111/mpp.13483)
Supplement: Supplementary file 3 — FIGURE S3. GbCML45 can interact with itself to form a dimer. (a) Yeast two‐hybrid assays showing interaction between GbCML45 and itself, but not happened in GbCML50 proteins. Yeast growth indicate interaction. (b) Bimolecular fluorescence complementation assays showing interaction between GbCML45 and itself in Nicotiana benthamiana epidermal cells. BES1‐cYFP and BIN2‐nYFP were used as positive control; bar = 100 μm. SDO (SD/−Trp); QDO (SD/−Trp/−Leu/−His/−Ade); BD‐53 + AD‐T7 (positive control); BD‐53 + AD‐Lam (negative control). [file MPP-25-e13483-s006.docx]

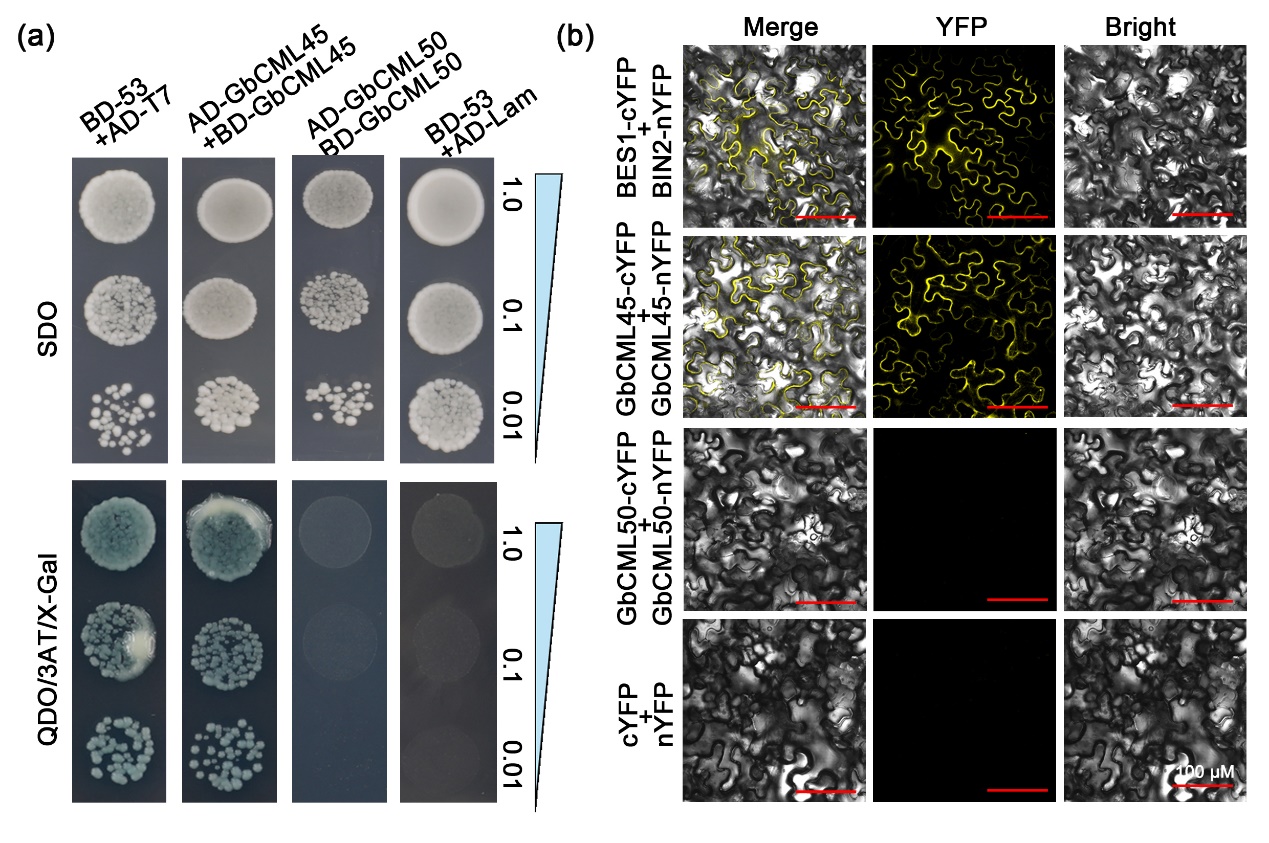


**Figure S3.** GbCML45 can interact with itself to form a dimer. (a) Yeast two-hybrid assays showing interaction between GbCML45 and itself, but not happened in GbCML50 proteins. Yeast growth indicate interaction. (b) BiFC assays showing interaction between GbCML45 and itself in tobacco epidermal cells. BES1-cYFP and BIN2-nYFP were used as positive control; bar = 100 µm. SDO (SD/-Trp); QDO (SD/-Trp/-Leu/-His/-Ade); BD-53 + AD-T7 (positive control); BD-53 + AD-Lam (Negtive control).
