# Supplementary material for: Positive roles of the Ca2+ sensors GbCML45 and GbCML50 in improving cotton Verticillium wilt resistance
Source: Mol Plant Pathol. 2024 Jun 3;25(6):e13483. doi: 10.1111/mpp.13483 (PMC11146148; doi:10.1111/mpp.13483)
Supplement: Supplementary file 4 — FIGURE S4. GbCML45 and GbCML50 overexpression Arabidopsis thaliana plants enhanced resistance to Verticillium dahliae infection, while reduced in the Atcml45 and Atcml 50 A. thaliana mutant plants. (a) Western blotting was used to detect the accumulation of GbCML45‐GFP and GbCML50‐GFP in different transgenic lines. Primary antibody of anti‐GFP mouse monoclonal antibody and the secondary antibody of horseradish peroxidase‐conjugated goat anti‐mouse IgG were used. (b) Disease symptoms of wild‐type (WT) and transgenic Arabidopsis plants ectopically expressing GbCML45 and GbCML50 plants at 15 days post‐inoculation (dpi) with V. dahliae. Photographs were taken at 15 dpi, and for each line at least 24 plants were observed. (c,d) Disease indices of wild‐type and GbCML45‐ and GbCML50‐overexpressing lines shown in (b). (e,f) Relative biomass of V. dahliae in GbCML45‐ and GbCML50‐overexpressing lines. (g) Disease symptoms of Atcml46 (GbCML45 orthologues) and Atcml49 (GbCML50 orthologues) mutant plants at 14 dpi with V. dahliae. (h) Disease index of Atcml46 and Atcml49 seedlings at 14 dpi with V. dahliae. (i) Relative biomass of V. dahliae in Atcml46 and Atcml49 seedlings. Data are means ± SD of three biological replicates (n = 3; ≥24 independent plants/biological replicate). Data was analysed using a two‐tailed Student’s ttest: **p < 0.01, ***p < 0.001. [file MPP-25-e13483-s007.docx]

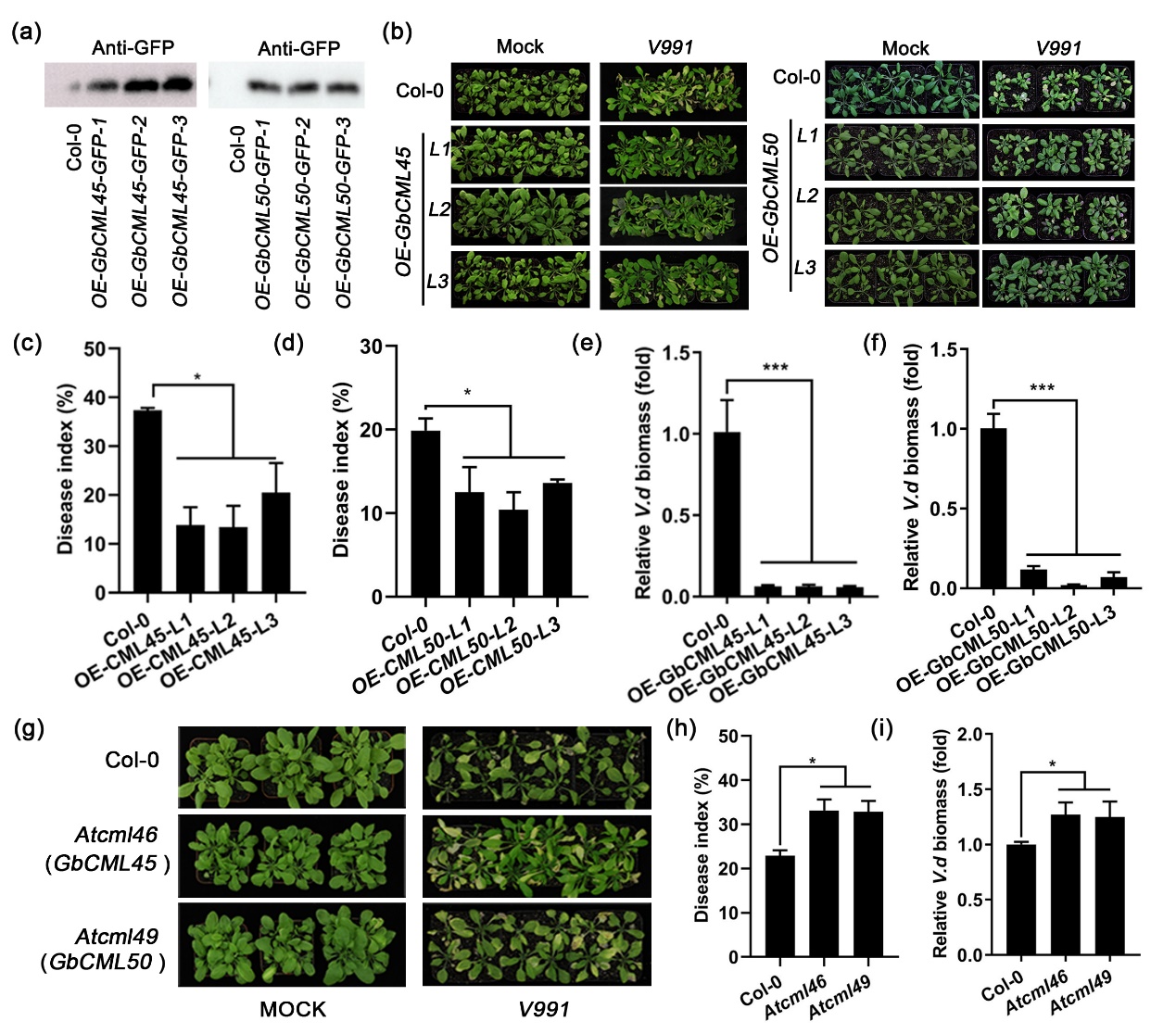


**Figure S4.** *GbCML45* and *GbCML50* overexpression *Arabidopsis* plants enhanced resistance to *Verticillium dahliae* infection, while reduced in the *Atcml45* and *Atcml 50 Arabidopsis* *thaliana* mutant plants. (a) Western blotting was used to detect the accumulation of GbCML45-GFP and GbCML50-GFP in different transgenic lines. Primary antibody of anti-GFP mouse monoclonal antibody and the secondary antibody of HRP-conjugated goat anti-mouse IgG were used. (b) Disease symptoms of wild-type (WT) and transgenic Arabidopsis plants ectopically expressing *GbCML45* and *GbCML50* plants at 15 dpi with *V. dahliae.* Pictures were photographed at 15 dpi, and for each line at least 24 plants were observed. (c-d) Disease indices of wild-type and *GbCML45*- and *GbCML50-*overexpressing lines shown in (b). (e-f) Relative biomass of *V. dahliae* in *GbCML45*- and *GbCML50-*overexpressing lines. (g) Disease symptoms of *Atcml46* (*GbCML45* orthologs) and *Atcml49* (*GbCML50* orthologs) mutant plants at 14 dpi with *V. dahliae*. (h) Disease index of *Atcml46* and *Atcml49* seedlings at 14 dpi with *V. dahliae.* (i) Relative biomass of *V. dahliae* in *Atcml46* and *Atcml49* seedlings. Data are means ± SD of three biological replicates (*n* = 3; ≥ 24 independent plants/biological replicate). Data was analyzed using a two-tailed Student’s *t*-test: **, *P* < 0.01; ***, *P* < 0.001.
