# Supplementary material for: Positive roles of the Ca2+ sensors GbCML45 and GbCML50 in improving cotton Verticillium wilt resistance
Source: Mol Plant Pathol. 2024 Jun 3;25(6):e13483. doi: 10.1111/mpp.13483 (PMC11146148; doi:10.1111/mpp.13483)
Supplement: Supplementary file 5 — FIGURE S5. Phylogenetic analysis of CMLs in cotton and Arabidopsis. A phylogenetic tree of CML proteins from Gossypium barbadense and Arabidopsis thaliana. The full‐length amino acid sequences of the CML proteins were aligned using ClustalX in MEGA 7.0. The unrooted tree was generated by the neighbour‐joining method (n = 1000 bootstraps). Cotton CMLs are coloured with red arrows and the Arabidopsis CMLs are in blue arrows. Different coloured solid circles indicate genes in different groups of Gossypium species or Arabidopsis. [file MPP-25-e13483-s005.docx]

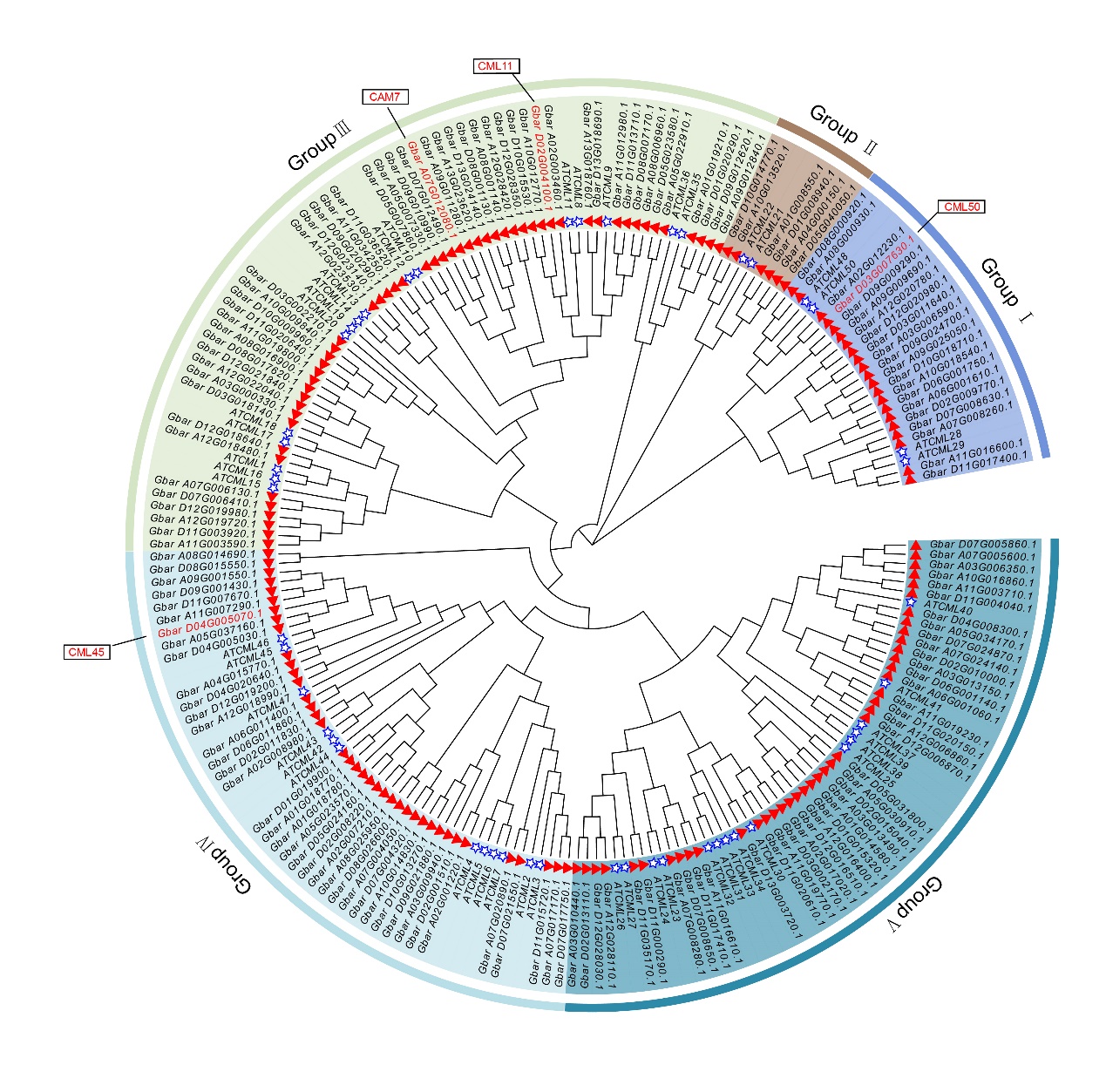


**Figure S5.** Phylogenetic analysis of CMLs in cotton and *Arabidopsis*. A phylogenetic tree of CMLs proteins from *G. barbadense* and *A. thaliana*. The full-length amino acid sequences of the CMLs proteins were aligned using Clustal X in MEGA7.0. The unrooted tree was generated by the neighbor-joining (NJ) method (n = 1000 bootstraps). Cotton a CMLs are colored with red arrows and the *Arabidopsis* CMLs are in blue arrows. Different-colored solid circles indicate genes in different groups of Gossypium species or Arabidopsis.
